# Straightforward Synthesis of Novel 1-(2′-α-*O*-D-Glucopyranosyl ethyl) 2-Arylbenzimidazoles

**DOI:** 10.3390/molecules17089887

**Published:** 2012-08-17

**Authors:** Natarajan Arumugam, Aisyah Saad Abdul Rahim, Shafida Abd Hamid, Hasnah Osman

**Affiliations:** 1 School of Pharmaceutical Sciences, Universiti Sains Malaysia, 11800 Minden, Penang, Malaysia; 2 Kulliyyah of Science, International Islamic University Malaysia (IIUM), Jalan Istana, Bandar Indera Mahkota, 25200 Kuantan, Pahang, Malaysia; 3 School of Chemical Sciences, Universiti Sains Malaysia, 11800 Minden, Penang, Malaysia

**Keywords:** 2-arylbenzimidazole, microwave-assisted synthesis, α-glycoside, glycosylation, glycomimetic

## Abstract

A series of novel 1-(2′-α-*O*-D-glucopyranosyl ethyl) 2-arylbenzimidazoles has been prepared via one-pot glycosylation of ethyl-1-(2'-hydroxyethyl)-2-arylbenzimidazole-5-carboxylate derivatives. Synthesis of the 2-arylbenzimidazole aglycones from 4-fluoro-3-nitrobenzoic acid was accomplished in four high-yielding steps. The reduction and cyclocondensation steps for the aglycone synthesis proceeded efficiently under microwave irradiation to afford the appropriate benzimidazoles in excellent yields within 2–3 min. Glycosylation of the hydroxyethyl aglycones with the perbenzylated 1-hydroxy- glucopyranose, pretreated with the Appel-Lee reagent, followed by catalytic hydrogenolysis delivered the desired 1-(2′-α-*O*-D-glucopyranosyl ethyl) 2-aryl-benzimidazoles in a simple and straightforward manner.

## 1. Introduction

Carbohydrate-protein interactions on cell surfaces mediate important biological processes and disease states, including cancer metastasis, inflammation, pathogenicity and Alzheimer’s disease [1,2,3,4,5,6,7]. Participating in such interactions are α-glycoside epitopes, found on bacteria, e.g., *Mycoplasma*, and on numerous mammalian oligosaccharides, for instance sialyl Lewis X (sLe^x^). The synthesis of oligosaccharide ligands as potential inhibitors, however, remains laboriously demanding [8,9,10].

Alternatively, these oligosaccharides can be simplified by retaining functional groups essential for key binding interactions and replacing the unwanted parts with heterocyclic scaffolds. This simplification strategy has led to the emergence of pharmaceutically relevant glycomimetics as potent inhibitors against new carbohydrate-based disease targets [8,9,11,12]. In recent years, considerable synthetic efforts were devoted to the preparation of glycosyl-modified heterocycles as sLe^x^ glycomimetics designed to inhibit selectin involvement in cancer metastasis and inflammation [4,5,9,11]. Additionally, several pyranosyl benzothiazoles and benzimidazoles have been found to inhibit α-glycosidases and glycogen phosphorylases, which are promising targets for treatment of diabetes mellitus [13,14,15,16].

Benzimidazoles are important heterocycles in medicinal chemistry with established clinical examples including the proton pump inhibitor omeprazole [17] and the antihelmintic albendazole [18,19]. Additionally, 1,2-difunctionalised benzimidazoles have shown diverse biological activities as antagonists against prostaglandin D2 [20] and angiotensin II receptors [21]. They have been prepared as guanine biomimetics that selectively suppress angiogenesis *in vitro* and *in vivo *[22]. Due to their biological significance, we became interested in the synthesis of substituted 2-arylbenzimidazoles as potential anti-infective and anti-proliferative agents. 

Recently, however, we encountered persistent problems with the solubility of such compounds during routine biological screening. To circumvent this solubility problem, we reasoned that by linking a sugar moiety to the 2-arylbenzimidazoles *via *a hydroxyethyl linker, not only could the sugar moiety modulate the solubility of the 2-arylbenzimidazoles, but it might also elicit novel pharmacological effects as an α-*O*-glycoside [12,23,24,25]. Furthermore, to the best of our knowledge, these α-*O*-glucosyl arylbenzimidazoles has not yet been reported. Thus, in this paper we describe, for the first time, a straightforward synthesis of novel 1-(2′-α-*O*-D-glucopyranosyl ethyl) 2-arylbenzimidazoles via one-pot glycosylation of hydroxyethyl arylbenzimidazole aglycones and 2,3,4,6-tetra-*O*-benzyl 1-hydroxylglucose employing the Appel-Lee reagent [26,27].

## 2. Results and Discussion

**Scheme 1:**
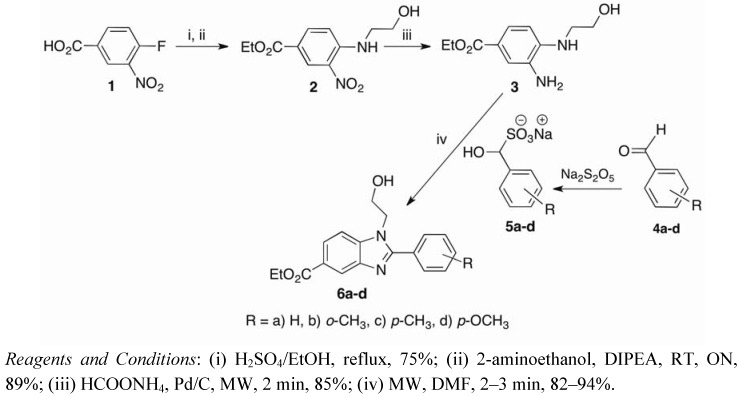
Synthesis of benzimidazole aglycones **6a**–**d**.

Our synthetic work started with esterification of the inexpensive precursor, 4-fluoro-3-nitrobenzoic acid (**1**). Treatment of the ester with 2-aminoethanol gave the amino intermediate **2**. Attempted reduction of the aromatic nitro group by refluxing with ammonium formate and 10% Pd/C for 3 h afforded the diamine **3** [28] in a modest 60% yield. After optimisation, microwave irradiation of the same reaction mixture at 100 °C for 2 min afforded **3** in a much improved 85% yield (Scheme 1).

**Figure 1:**
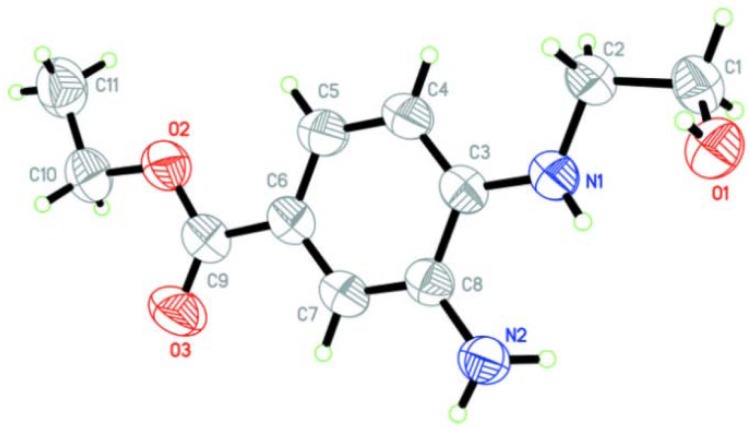
ORTEP digram of **3** (CCDC 788495).

This diamine was found to be stable at room temperature, unlike other alkylated phenylenediamine derivatives that we had prepared previously; these turned brown and decomposed, even when stored at 5–10 °C. The stability of the amino derivative **3** was possibly due to intramolecular hydrogen bonding between the OH and NH_2_ groups, as apparent from single X-ray crystallographic analysis (Figure 1) [28].

Next we turned our attention to the synthesis of 2-arylbenzimidazoles **6a**–**d**. These are typically prepared via condensation reactions of phenylenediamines with the corresponding acids or aldehydes [29,30]. Harsh, dehydrating conditions are often a requisite in cyclocondensation reactions with aromatic acids. More facile condensations can be achieved via arylaldehydes by employing oxidative reagents, such as Cu(OAc)_2_, air, 1,4-benzoquinone, I_2_/KI and sodium metabisulfite [29,31,32,33]. After taking into consideration previous reports and the availability of commercial benzaldehydes, we initially attempted the cyclocondensation with the diamine **3**, aromatic aldehydes and sodium metabisulfite in one pot as reported by Navarrete-Vázquez *et al.* [33] under conventional heating conditions. The one-pot cyclocondensation failed to afford the benzimidazole products. Upon heating under microwave conditions, the same reaction gave multiple spots on TLC, but we were unable to isolate the desired benzimidazoles. Due to the unsuccessful attempts at the one-pot cyclocondensation reaction, we then decided to react the diamine **3** with the metabisulfite adducts of arylaldehydes **5a**–**d** [33,34]. The conventional reaction conditions (refluxing in DMF) initially suffered from long reaction times and afforded only moderate yields of the desired benzimidazoles **6a**–**d**. When the same reactions were performed under optimised microwave conditions [33,35], the benzimidazole aglycones **6a**–**d** were obtained in excellent 82–94% yields within 2–3 min (Table 1) using minimal solvent (0.5–1 mL). Our results show that using microwaves as a heating source not only improves yields of the desired benzimidazoles, but it also brings about tremendous reductions in reaction times and the amount of solvent required.

**Table 1:** Influence of microwave irradiation and conventional heating on the synthesis of benzimidazole derivatives **6a**–**d**.

Entry	Products	R	Conventional heating		Microwave heating	
			Time (h)	Yield (%)	Time (min)	Yield (%)
1	**6a**	H	3.5	62	3	88
2	**6b**	o-CH_3_	3	65	2.5	82
3	**6c**	*p*-CH_3_	2.5	67	2	94
4	**6d**	*p*-OCH_3_	3	60	2	89

**Figure 2:**
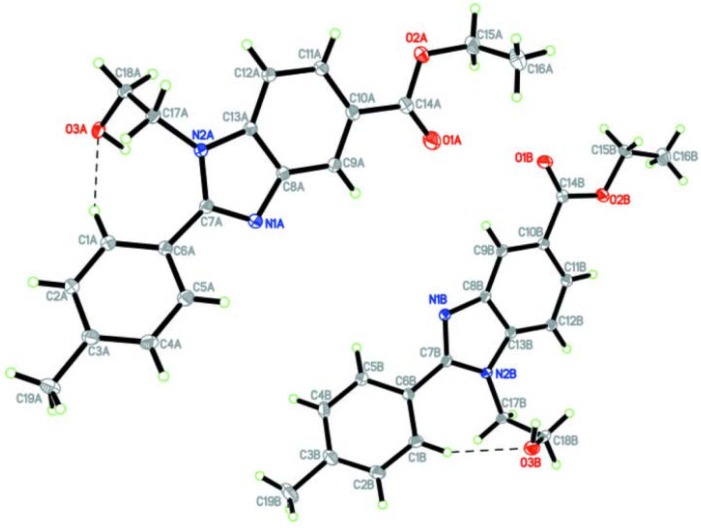
ORTEP digram of **6c** (CCDC 786546).

The ^1^H-NMR spectrum of benzimidazole **6c** showed the loss of the broad singlet NH_2_ peak at δ 4.60–4.85, which corroborates with the formation of the imine (C=N) that resonated at δ 156.1 in the ^13^C-NMR spectrum. High resolution mass spectrometry data revealed a peak at *m/z *= 325.1549 (M+H requires 325.1547), which corresponds to **6c**. Single crystal X-ray analysis [36] confirmed the structure of **2c** (Figure 2). Arylbenzimidazoles derivatives **6a**, **6b** and **6d** showed similar spectroscopic patterns.

With the alcohols **6a**–**d** in hand, we next required a suitable glycosylation method to furnish the α-*O*-glycosyl benzimidazoles in a facile manner. Derivatives of α*-O-*glycosides can be accessed in a number of ways [37,38], one of the most efficient being the established *in situ *anomerisation procedure which employs a 1-bromo sugar as the glycosyl donor. The tedious and costly preparation of glycosyl bromides coupled with the corrosive nature of HBr gas prompted the search for alternative methods to generate the desired bromides *in situ*. Several one-pot reactions were reported to furnish glucosyl [39], galactosyl [40] and fucosyl [41] intermediates in moderate to good yields. Recently, Shingu *et al*. described a practical one pot α-glycosylation method based on the Appel-Lee reaction utilizing PPh_3_ and CBr_4_ [23,42].

**Scheme 2:**
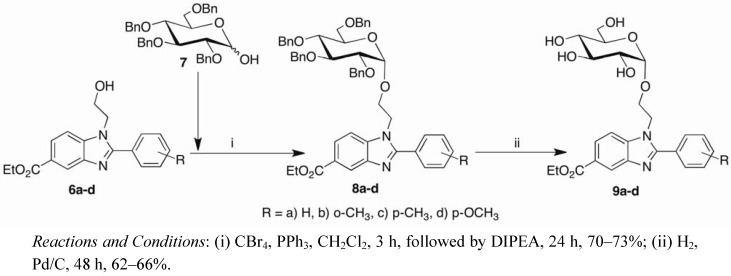
Synthesis of α-*O-*glucosyl benzimidazoles **9a**–**d**.

Motivated by these findings, we attempted the glycosylation of the alcohols **6a**–**d** by pre-treating commercially available 2,3,4,6-tetra-*O*-benzyl-D-glucopyranose (**7**) with the Appel-Lee reagents for 3 h. This resulted in the *in situ *formation of glycosyl bromide, which underwent glycosylation with the alcohols **6a**–**d** after a further 24 h. This one-pot glycosylation step yielded the perbenzylated α-*O-*glucosyl benzimidazoles **8a**–**d** in 70–73% yields. Finally, catalytic hydrogenolysis step afforded the target hydroxyl sugars **9a**–**d** (Scheme 2).

Via the optimised conditions, the glycosylated products were obtained as a mixture of α:β anomers (95:5), which is comparable to the ratios reported by Shingu [42]. The ^1^H-NMR of the isolated anomer **8c** showed the α-proton appearing at δ 4.60 as a doublet (*J* = 3.3 Hz). This small *J* value strongly indicated the successful formation of the desired α-*O-*glycosidic linkage between the glucopyranoside moiety and 2'-hydroxyethyl 2-arylbenzimidazole scaffold. Further confirmation came from the ^1^H-NMR spectrum and HRMS of the deprotected sugar **9c**. Absence of benzylic protons in **9c** revealed the characteristic α-proton (δ 4.61–4.68), and the HRMS showed the molecular peak at 487.2077 (M+H requires 487.2075) corresponding to the hydroxyl sugar **9c**. The structures of the remaining α-*O*-glucosylated arylbenzimidazole derivatives were established spectroscopically and corroborated with **8c** and **9c**.

## 3. Experimental

### 3.1. General

All ^1^H- and ^13^C-NMR spectra were recorded on Bruker 300 and 400 MHz instruments in CDCl_3_ and DMSO-d_6_. High resolution mass spectrometry (HRMS) measurements of the benzimidazole derivatives were acquired on an Agilent 6520 Quadrupole Time of Flight Mass Spectrometer (Agilent Technologies, Santa Clara, CA, USA) operating in the MS mode. Microwave-assisted syntheses were performed in CEM Discover™ microwave synthesizer. Melting points were measured on a Stuart SMP10 instrument and are uncorrected. Preparative thin layer chromatography (PLC) was performed using Merck 60 GF_254_ silica gel coated (1 mm) on glass plates (20 × 20 cm). TLC experiments were performed on alumina-backed silica gel 40 F254 plates (Merck, Darmstadt, Germany). Visualisation of TLC plates was performed under UV light and aided by KMnO_4_, iodine staining and 2% H_2_SO_4_ in EtOH (charcoal staining for sugar). All commercially available starting materials and solvents are from Sigma Aldrich, Acros and Merck; they were used without further purification. 

*Synthesis of ethyl 3-amino-4-(2-hydroxyethylamino)benzoate* (**3**). A solution of 4-fluoro-3-nitrobenzoic (**1**, 10 g, 0.054 mol) was refluxed in EtOH (100 mL) and conc. H_2_SO_4_ (4 mL) for 8 h. After completion of reaction, evidenced by TLC analysis, the excess solvent was removed under reduced pressure. The aqueous layer was extracted with EtOAc (50 × 2 mL). Upon washing with 10% NaHCO_3 _(100 mL), the combined organic layer was dried over Na_2_SO_4_ and concentrated. Crystallisation of the crude product with hot hexane yielded the desired ethyl ester as colourless crystals (8.6 g). A portion of the benzoate (2.0 g, 9.2 mmol) in dichloromethane (25 mL) was added to a solution of ethanolamine (0.687 g, 14.0 mmol) and DIPEA (1.45 g, ~2 mL, 11.2 mmol) in dichloromethane (25 mL). The reaction mixture was stirred overnight at room temperature, then washed with water (20 mL × 2) and 10% Na_2_CO_3_ (20 mL × 2). The dichloromethane layer was collected, dried over Na_2_SO_4_ and removed under reduced pressure to afford the amino compound **2** (2.07 g, 89%). The amine **2** was used in the next step without purification, thus to a solution of the amine **2** (500 mg, 1.96 mmol) in EtOH (4 mL) was added HCOONH_4_ (430 mg, 6.82 mmol) and 10% Pd/C (250 mg, 2.34 mmol). The reaction mixture was irradiated using a CEM DiscoverÔ microwave synthesizer for 2 min at 100 °C. After completion, the mixture was filtered through a bed of Celite and the filtrate evaporated under reduced pressure to afford the title product **3** as white crystals (0.45 g, 85%). Mp 116*–*118 °C; ^1^H-NMR (DMSO-d_6_, 300 MHz): δ 1.26 (t, *J =* 6.3 Hz, CH_3_, 3H), 3.16*–*3.21 (m, CH_2_, 2H), 3.61*–*3.69 (m, CH_2_, 2H), 4.18 (q, *J =* 7.2 Hz, OCH_2_, 2H), 4.73 (br s, NH_2_, 2H), 5.17*–*5.21 (m, NH, 1H), 6.46 (d, *J =* 8.1 Hz, 1H), 7.18*–*7.24 (m, 2H) ppm. ^13^C-NMR (DMSO-d_6_, 75 MHz): δ 15.3, 46.3, 60.1, 60.3, 108.7, 117.7, 134.9, 141.5, 167.2 ppm. ESI-MS *m/z* 225.2 (M+1). CCDC 788495 contains the supplementary crystallographic data for structure **3**.

### 3.2. General Procedure for the Microwave-Assisted Synthesis of Benzimidazoles ***6a**–**d***

The metabisulfite adduct **5** (2.0 eq.) was added to a solution of 3-amino-4-(2-hydroxyethylamino) benzoate (**3**, 1.0 eq.) in DMF (0.5–1 mL). The reaction mixture was heated under microwave conditions at 130 °C for 2 min. After completion, the mixture was diluted with EtOAc (10 mL) and washed with H_2_O (10 mL). The organic layer was collected, dried over Na_2_SO_4_ and evaporated *in vacuo* to yield a crude residue, which was recrystallised from EtOAc to afford the desired benzimidazole as colourless crystals.

*Ethyl 2-phenyl-1-(2-hydroxyethyl)-1H-benzimidazole-5-carboxylate* (**6a**). Colourless crystals (0.97 g, 88%). Mp 123*–*125 °C; IR (KBr) 3400, 1637, 1265, 740 cm*^–^*^1^; ^1^H-NMR (CDCl_3_, 300 MHz): δ 1.46 (t, CH_3_, 3H), 4.15*–*4.29 (m, 2CH_2_, 4H), 4.41 (q, CH_2_, 2H), 6.05*–*6.18 (s, 1H), 7.20*–*7.84 (m, 8H) ppm. ^13^C-NMR (CDCl_3_, 75 MHz): δ 14.8, 47.5, 60.7, 61.2, 110.0, 121.4, 124.3, 125.1, 128.8, 129.0, 130.2, 130.4, 138.3, 141.6, 156.1, 167.1 ppm. HRMS (ESI/Q-TOF): *m/z* calcd for C_18_H_18_N_2_O_3_ (M+H), 311.1390; found 311.1391.

*Ethyl 1-(2-hydroxyethyl)-2-o-tolyl-1H-benzimidazole-5-carboxylate* (**6b**). White crystals (0.35 g, 82%). Mp 119*–*121 °C; IR (KBr) 3398, 1641, 1273, 752 cm*^–^*^1^; ^1^H-NMR (CDCl_3_, 300 MHz): δ 1.41 (t, CH_3_, 3H), 2.12 (s, CH_3_, 3H), 3.69 (t, *J* = 5.7 Hz, CH_2_, 2H), 4.04 (t, *J* = 5.7 Hz, CH_2_, 2H), 4.39 (q, CH_2_, 2H), 7.17*–*7.42 (m, 5H), 7.92*–*7.95 (dd, *J* = 8.5, 1.6 Hz, 1H), 8.38 (d, *J* = 1.2 Hz, 1H) ppm. ^13^C-NMR (CDCl_3_, 75 MHz): δ 14.4, 19.6, 46.6, 60.3, 61.0, 110.2, 121.7, 124.2, 124.7, 125.7, 129.2, 130.1, 130.2, 130.5, 137.9, 138.0, 142.0, 155.3, 167.2 ppm. HRMS (ESI/Q-TOF): *m/z* calcd for C_19_H_20_N_2_O_3_ (M+H), 325.1547; found 325.1548.

*Ethyl 1-(2-hydroxyethyl)-2-p-tolyl-1H-benzimidazole-5-carboxylate* (**6c**). Colourless crystals (1.0 g, 94%). Mp 138*–*140 °C; IR (KBr) 3377, 1639, 1275, 749 cm*^–^*^1^; ^1^H-NMR (CDCl_3_, 300 MHz): δ 1.47 (t, *J* = 7.2 Hz, CH_3_, 3H), 2.38 (s, CH_3_, 3H), 4.18*–*4.30 (m, 2CH_2_, 4H), 4.41 (q, *J* = 7.2 Hz, OCH_2_, 2H), 6.25*–*6.40 (s, 1H), 7.00 (d, *J* = 8.1 Hz, 2H), 7.21 (d, *J* = 8.4 Hz, 1H), 7.59 (d, *J* = 8.1 Hz, 2H), 7.71 (d, *J* = 8.7 Hz, 1H), 7.78 (s, 1H) ppm. ^13^C-NMR (CDCl_3_, 75 MHz): δ 14.8, 21.8, 47.5, 60.7, 61.2, 109.9, 121.2, 124.1, 125.0, 126.1, 129.4, 130.3, 138.3, 140.2, 141.5, 156.1, 167.0 ppm. HRMS (ESI/Q-TOF): *m/z* calcd for C_19_H_20_N_2_O_3_ (M+H), 325.1547; found 325.1549. CCDC 786546 contains the supplementary crystallographic data for structure **6c**.

*Ethyl 1-(2-hydroxyethyl)-2-(4-methoxyphenyl)-1H-benzimidazole-5-carboxylate* (**6d**). Colourless crystals (0.94 g, 89%). Mp 120*–*122 °C; IR (KBr) 3402, 1642, 1265, 740 cm*^–^*^1^; ^1^H-NMR (CDCl_3_, 300 MHz): δ 1.47 (t, CH_3_, 3H), 3.83 (s, OCH_3_, 3H), 4.22*–*4.30 (m, 2CH_2_, 4H), 4.41 (q, CH_2_, 2H), 6.71 (d, *J* = 9.0 Hz, 2H), 7.19 (d, *J* =8.4 Hz, 1H), 7.67*–*7.73 (m, 4H) ppm. ^13^C-NMR (CDCl_3_, 75 MHz): δ 14.9, 47.5, 55.6, 60.7, 61.2, 109.7, 114.1, 121.0, 121.2, 124.0, 125.0, 132.0, 138.1, 141.4, 156.0, 161.1, 167.0 ppm. HRMS (ESI/Q-TOF): *m/z* calcd for C_19_H_20_N_2_O_4_ (M+H), 341.1496; found 341.1499.

### 3.3. General Procedure for the Synthesis of 1-(2′-α-O-D-glucopyranosyl ethyl) 2-arylbenzimidazoles ***8a**–**d***

A solution of 2,3,4,6-tetra-*O*-benzyl-D-glucopyranose (**7**, 1.0 eq.), PPh_3_ (3.0 eq.) and CBr_4_ (3.0 eq.) in CH_2_Cl_2_ (3 mL) was stirred for 3 h under N_2_ atmosphere at room temperature. Upon completion of reaction as evidenced by TLC analysis, a solution of DIPEA (2.5 eq.) followed by substituted benzimidazole **6** (3.0 eq.) were added to the reaction mixture, which was stirred at room temperature under N_2_ atmosphere for a further 24 h. The crude reaction mixture was purified by column chromatography using EtOAc-hexanes (3:7) to afford the product as a semi-solid.

*Ethyl 1-[2′-α-O-D-(2,3,4,6-tetra-O-benzylglucopyranosyl)ethyl]-2-phenyl-1H-benzimidazole-5-carboxylate* (**8a**). Isolated as low melting solid (0.27 g, 70%). IR (film) 3409, 1611, 1265, 740 cm*^–^*^1^; ^1^H-NMR (CDCl_3_, 300 MHz): δ 1.44 (t, CH_3_, 3H), 3.23*–*3.30 (m, H-2), 3.32*–*3.36 (m, H-4), 3.46*–*3.51 (m, CH_2_, 2H), 3.54*–*3.60 (m, H-6_a_), 3.68*–*3.73 (m, H-6_b_), 3.76*–*3.82 (m, H-5), 3.98*–*4.06 (m, H-3), 4.37*–*4.41 (m, CH_2_, 2H), 4.42*–*4.45 (m, CH_2_, 2H), 4.59 (d, *J* = 3.3 Hz, H-1), 4.46*–*4.94 (m, PhCH_2_, 8H), 7.11*–*8.59 (m, Ar-H, 28H) ppm. ^13^C-NMR (CDCl_3_, 75 MHz): δ 14.3, 44.6, 60.8, 65.9, 68.2, 70.5, 73.3, 73.4, 74.7, 75.7, 76.6, 79.8, 81.7, 97.6, 110.2, 122.2, 124.4, 124.8, 127.6, 127.7, 127.8, 127.9, 128.0, 128.3, 128.4, 128.8, 129.1, 129.2, 129.8, 130.1, 138.2, 138.2, 142.6, 155.8, 167.0 ppm. HRMS (ESI/Q-TOF): *m/z* calcd for C_52_H_52_N_2_O_8_ (M+H), 833.3797; found 833.3799.

*Ethyl 1-[2'-α-O-D-(2,3,4,6-tetra-O-benzylglucopyranosyl)ethyl]-2-o-tolyl-1H-benzimidazole-5-carboxylate* (**8b**). Isolated as low melting solid (0.20 g, 73%). IR (film) 3408, 1621, 1266 cm*^–^*^1^; ^1^H-NMR (CDCl_3_, 300 MHz): δ 1.41 (t, CH_3_, 3H), 2.23 (s, CH_3_, 3H), 3.23*–*3.30 (m, H-2), 3.35*–*3.39 (m, H-4), 3.45*–*3.49 (m, CH_2_, 2H), 3.51*–*3.61 (m, H-6_a_), 3.76*–*3.80 (m, H-6_b_), 3.81*–*3.87 (m, H-5), 4.18*–*4.25 (m, H-3), 4.27*–*4.37 (m, CH_2_, 2H), 4.38*–*4.44 (m, CH_2_, 2H), 4.53 (d, *J* = 3.3 Hz, H-1), 4.46*–*4.97 (m, PhCH_2_, 8H), 7.10*–*8.58 (m, 27H) ppm. ^13^C-NMR (CDCl_3_, 75 MHz): δ 14.8, 20.1, 44.3, 61.2, 66.5, 68.7, 71.0, 73.7, 73.8, 75.2, 76.1, 80.2, 82.1, 98.1, 110.7, 122.7, 124.7, 125.3, 126.3, 128.0, 128.1, 128.2, 128.3, 128.4, 128.7, 128.8, 129.9, 130.6, 130.9, 138.1, 138.3, 138.5, 138.6, 139.1, 143.1, 155.6, 167.4 ppm. HRMS (ESI/Q-TOF): *m/z* calcd for C_53_H_54_N_2_O_8_ (M+H), 847.3953; found 847.3953.

*Ethyl 1-[2′-α-O-D-(2,3,4,6-tetra-O-benzylglucopyranosyl)ethyl]-2-p-tolyl-1H-benzimidazole-5-carboxylate* (**8c**). Isolated as low melting solid (0.17 g, 72%). IR (film) 3392, 1645, 1261, 750 cm*^–^*^1^; ^1^H-NMR (CDCl_3_, 300 MHz): δ 1.41 (t, *J* = 7.1 Hz, CH_3_, 3H), 2.42 (s, CH_3_, 3H), 3.24*–*3.31 (m, H-2, 1H), 3.32*–*3.38 (m, H-4, 1H), 3.47*–*3.50 (m, CH_2_, 2H), 3.51*–*3.55 (m, H-6_a_, 1H), 3.65*–*3.73 (m, H-6_b_, 1H), 3.75*–*3.82 (m, H-5, 1H), 3.98*–*4.05 (m, H-3, 1H), 4.37*–*4.39 (m, CH_2_, 2H), 4.40*–*4.44 (m, CH_2_, 2H), 4.60 (d, *J* = 3.3 Hz, H-1, 1H), 4.45*–*4.94 (m, PhCH_2_, 8H), 7.10*–*7.35 (m, Ar-H, 22H), 7.54 (d, *J* = 8.7 Hz, 1H), 7.74 (d, *J* = 8.1 Hz, 2H), 8.04*–*8.08 (dd, *J* = 8.1, 1.5 Hz, 1H), 8.57 (d, *J* = 1.5 Hz, 1H) ppm. ^13^C-NMR (CDCl_3_, 75 MHz): δ 14.8, 21.9, 45.0, 61.3, 66.4, 68.6, 70.9, 73.6, 73.7, 73.8, 75.2, 76.1, 80.2, 82.1, 98.0, 110.6, 122.6, 124.7, 125.5, 127.2, 128.0, 128.1, 128.2, 128.3, 128.4, 128.7, 128.8, 129.9, 130.0, 138.1, 138.3, 138.6, 139.1, 139.3, 140.7, 143.0, 156.4, 167.5 ppm. HRMS (ESI/Q-TOF): *m/z* calcd for C_53_H_54_N_2_O_8_ (M+H), 847.3953; found 847.3953.

*Ethyl 1-[2'-α-O-D-(2,3,4,6-tetra-O-benzylglucopyranosyl)ethyl]-2-p-methoxyphenyl-1H-benzimidazole-5-carboxylate* (**8d**). Isolated as low melting solid (0.28 g, 71%). IR (film) 3421, 1630, 1265, 740 cm*^–^*^1^; ^1^H-NMR (CDCl_3_, 300 MHz): δ 1.40 (t, CH_3_, 3H), 3.24*–*3.30 (m, H-2), 3.32*–*3.36 (m, H-4), 3.46*–*3.48 (m, CH_2_, 2H), 3.49*–*3.54 (m, H-6_a_), 3.70*–*3.78 (m, H-6_b_), 3.81 (s, OCH_3_, 3H), 3.82*–*3.84 (m, H-5), 3.98*–*4.04 (m, H-3), 4.35*–*4.39 (m, CH_2_, 2H), 4.40*–*4.43 (m, CH_2_, 2H), 4.60 (d, *J* = 3.6 Hz, H-1), 4.46*–*4.92 (m, PhCH_2_, 8H), 6.98 (d, *J* = 8.7 Hz, 2H), 7.10*–*7.33 (m, 20H), 7.51 (d, *J* = 8.4 Hz, 1H), 7.80 (d, *J* = 9 Hz, 2H), 8.01*–*8.05 (dd, *J* = 1.5, 8.4 Hz, 1H), 8.54 (d, *J* = 1.5 Hz, 1H) ppm. ^13^C-NMR (CDCl_3_, 75 MHz): δ 14.8, 45.0, 55.7, 61.2, 66.3, 68.6, 70.9, 73.7, 73.8, 75.2, 76.1, 80.3, 82.2, 98.0, 110.4, 114.7, 122.4, 124.6, 125.5, 127.9, 128.0, 128.1, 128.2, 128.3, 128.4, 128.7, 128.8, 131.7, 138.1, 138.6, 139.1, 139.4, 143.0, 156.2, 161.5, 167.5 ppm. HRMS (ESI/Q-TOF): *m/z* calcd for C_19_H_20_N_2_O_3_ (M+H), 863.3902; found 863.3901.

### 3.4. General Procedure for the Catalytic Hydrogenolysis

A solution of perbenzylated glucopyranosyl arylbenzimidazole **8** (200 mg, 0.23 mmol) in MeOH (10 mL) was hydrogenated in the presence of 10% Pd/C (100 mg, 0.93 mmol) at room temperature for 48 h. The reaction mixture was filtered through a bed of Celite and washed with MeOH (10 mL × 3). The solvent was removed *in vacuo* to afford a crude residue which was purified by column chromatography in CHCl_3_–MeOH (9:1) to give the desired product as a light yellow semisolid.

*Ethyl 1-[2'-α-O-D-glucopyranosyl ethyl]-2-phenyl-1H-benzimidazole-5-carboxylate *(**9a**). Isolated as light yellow semisolid (0.14 g, 62%). IR (film) 3411, 1635, 1275, 750 cm^–1^; ^1^H-NMR (CDCl_3_, 300 MHz): δ 1.34 (t, CH_3_, 3H), 2.64 (d, *J* = 9.3 Hz, H-4), 3.15 (d, *J* = 8.1 Hz, H-2), 3.24–3.30 (m, CH_2_, 2H), 3.34–3.38 (m, H-3), 3.39–3.42 (m, H-5), 3.69–3.77 (m, H-6_a_), 3.93–4.03 (m, H-6_b_), 4.29 (q, CH_2_, 2H), 4.33–4.49 (m, CH_2_, 2H), 4.57–4.64 (m, H-1), 7.37–7.51 (m, 4H), 7.75–7.76 (m, 2H), 7.92–7.94 (m, 1H), 8.43 (s, 1H) ppm. ^13^C-NMR (CDCl_3_, 75 MHz): δ 14.7, 44.8, 60.9, 61.5, 65.6, 69.3, 71.8, 72.2, 74.1, 99.0, 110.7, 122.3, 124.8, 125.4, 129.3, 129.7, 130.1, 130.7, 139.0, 142.5, 156.4, 167.6 ppm. HRMS (ESI/Q-TOF): *m/z *calcd for C_24_H_28_N_2_O_8_ (M+H), 473.1919; found 473.1919.

*Ethyl 1-[2′-α-O-D-glucopyranosyl ethyl]-2-o-tolyl-1H-benzimidazole-5-carboxylate *(**9b**). Isolated as pale yellow semisolid (0.11 g, 66%). IR (film) 3404, 1635, 1265, 744 cm^–1^; ^1^H-NMR (CDCl_3_, 300 MHz): δ 1.37 (t, CH_3_, 3H), 2.16 (s, CH_3_, 3H), 2.71 (d, *J* = 9.3 Hz, H-4), 3.22–3.26 (m, H-2), 3.27–3.30 (m, CH_2_, 2H), 3.41–3.43 (m, H-3), 3.44–3.51 (m, H-5), 3.58–3.62 (m, H-6_a_), 3.80–3.91 (m, H-6_b_), 4.18–4.28 (m, CH_2_, 2H) 4.34 (q, CH_2_, 2H), 4.60 (d, *J *= 2.7 Hz, H-1), 7.31–7.39 (m, 3H), 7.50–7.57 (m, 2H), 7.98–8.02 (dd, *J* = 8.4, 1.2 Hz, 1H), 8.47 (d, *J* = 1.2 Hz, 1H) ppm. ^13^C-NMR (CDCl_3_, 75 MHz): δ 14.7, 20.0, 44.3, 61.2, 61.4, 66.2, 69.6, 71.9, 72.2, 74.1, 99.1, 110.9, 122.4, 124.8, 125.3, 126.5, 129.4, 130.8, 131.0, 138.1, 138.5, 142.5, 155.8, 167.8 ppm. HRMS (ESI/Q-TOF): *m/z *calcd for C_25_H_30_N_2_O_8_ (M+H), 487.2075; found 487.2079.

*Ethyl 1-[2'-α-O-D-glucopyranosyl ethyl]-2-p-tolyl-1H-benzimidazole-5-carboxylate *(**9c**). Isolated as pale yellow semisolid (0.10 g, 65%). IR (film) 3411, 1608, 1420, 1265, 740 cm^–1^; ^1^H-NMR (CDCl_3_, 400 MHz): δ 1.36 (t, *J *= 7.2 Hz, CH_3_, 3H), 2.32 (s, CH_3_, 3H), 2.62 (d, *J *= 9.2 Hz, H-4), 3.17 (d, *J *= 11.2 Hz, H-2), 3.26–3.35 (m, CH_2_, 2H), 3.36–3.40 (m, H-3), 3.41–3.47 (m, H-5), 3.71–3.73 (m, H-6_a_), 3.95–4.03 (m, H-6_b_), 4.31 (q, *J *= 7.2 Hz, CH_2_, 2H), 4.37–4.43 (m, CH_2_, 1H), 4.47–4.57 (m, CH_2_, 1H), 4.61–4.68 (m, H-1), 7.25–7.28 (m, 2H), 7.47 (d, *J *= 8.4 Hz, 1H), 7.64 (d, *J *= 8.0 Hz, 2H), 7.94 (d, *J *= 8.8 Hz, 1H), 8.42 (s, 1H) ppm. ^13^C-NMR (CDCl_3_, 75 MHz): δ 14.7, 21.8, 44.8, 60.9, 61.5, 65.6, 69.4, 71.8, 72.2, 74.2, 99.0, 110.7, 122.2, 124.8, 125.4, 126.7, 130.0, 139.1, 141.0, 142.4, 156.6, 167.7 ppm. HRMS (ESI/Q-TOF): *m/z *calcd for C_25_H_30_N_2_O_8_ (M+H), 487.2075; found 487.2077.

*Ethyl 1-[2'-α-O-D-glucopyranosyl ethyl]-2-p-methoxyphenyl-1H-benzimidazole-5-carboxylate *(**9d**)*.* Isolated as light yellow semisolid (0.06 g, 64%). IR (film) 3402, 1616, 1265, 747 cm^–1^; ^1^H-NMR (CDCl_3_, 300 MHz): δ 1.35 (t, CH_3_, 3H), 2.61 (d, *J* = 9.0 Hz, H-4), 3.13 (d, *J *= 10.5 Hz, H-2), 3.21–3.30 (m, CH_2_, 2H), 3.33–3.37 (m, H-3), 3.38–3.46 (m, H-5), 3.72 (s, OCH_3_, 3H), 3.73–3.79 (m, H-6_a_), 3.94–4.08 (m, H-6_b_), 4.30 (q, CH_2_, 2H), 4.38–4.57 (m, CH_2_, 2H), 4.59–4.64 (m, H-1), 6.95 (d, *J* = 8.7 Hz, 2H), 7.42 (d, *J *= 8.4 Hz, 1H), 7.70 (d, *J *= 8.4 Hz, 2H), 7.91 (d, *J *= 8.4 Hz, 1H), 8.39 (s, 1H) ppm. ^13^C-NMR (CDCl_3_, 75 MHz): δ 14.7, 44.8, 55.7, 60.8, 61.4, 65.5, 69.3, 71.8, 72.2, 74.1, 99.0, 110.5, 114.8, 121.8, 122.0, 124.6, 125.3, 131.6, 139.0, 142.4, 156.4, 161.4, 167.7 ppm. HRMS (ESI/Q-TOF): *m/z *calcd for C_25_H_30_N_2_O_9_ (M+H), 503.2024; found 503.2025.

## 4. Conclusions

We have described a simple and straightforward synthesis of a series of novel α-*O*-glucopyranosyl arylbenzimidazoles using the Appel-Lee reagents. The synthesis of the glycosyl acceptors, 2-arylbenzimidazoles **6a**–**d**, was accomplished in four, high-yielding steps from the inexpensive precursor 4-fluoro-3-nitrobenzoic acid. Optimised microwave conditions for the reduction and cyclocondensation steps afforded the 2-arylbenzimidazole aglycones in high yields (82%–94%) and short reaction times (2–3 min) using reduced amount of solvent. This facile approach would allow rapid preparation of similar glycosylated benzimidazoles, which will be further investigated under both conventional and microwave conditions. Bioactivity studies of these glycosyl benzimidazoles will be reported in due course. 

## Supplementary Materials

Supplementary materials can be accessed at: http://www.mdpi.com/1420-3049/17/8/9887/s1.

## References

[1] Varki A. (1993). Biological roles of oligosaccharides: All of the theories are correct. Glycobiology.

[2] Gabius H.-J. (2008). Glycans: Bioactive signals decoded by lectins. Biochem. Soc. Trans..

[3] Varki A. (2006). Nothing in glycobiology makes sense, except in the light of evolution. Cell.

[4] Unger F.M. (2001). The chemistry of oligosaccharide ligands of selectins: significance for the development of new immunomodulatory medicines. Adv. Carbohydr. Chem. Biochem..

[5] Simanek E.E., McGarvey G.J., Jablonowski J.A., Wong C.-H. (1998). Selectin-carbohydrate interactions: From natural ligands to designed mimics. Chem. Rev..

[6] Sato N., Oizumi T., Kinbara M., Sato T., Funayama H., Sato S., Matsuda K., Takada H., Sugawara S., Endo Y. (2010). Promotion of arthritis and allergy in mice by aminoglycoglycerophospholipid, a membrane antigen specific to *Mycoplasma fermentans*. FEMS Immunol. Med. Microbiol..

[7] Kawahito Y., Ichinose S., Sano H., Tsubouchi Y., Kohno M., Yoshikawa T., Tokunaga D., Hojo T., Harasawa R., Nakano T. (2008). Mycoplasma fermentans glycolipid-antigen as a pathogen of rheumatoid arthritis. Biochem. Biophys. Res. Commun..

[8] Kaila N., Thomas B.E. (2002). Design and synthesis of sialyl Lewis(x) mimics as E- and P-selectin inhibitors. Med. Res. Rev..

[9] Chhabra S.R., Rahim A.S.A., Kellam B. (2003). Recent progress in the design of selectin inhibitors. Mini-Rev. Med. Chem..

[10] Saotome C., Kanie O., Wong C.-H. (2003). Synthetic methodologies. Carbohydrate-Based Drug Discovery.

[11] Ernst B., Magnani J.L. (2009). From carbohydrate leads to glycomimetic drugs. Nat. Rev. Drug Discov..

[12] Cipolla L., La Ferla B., Airoldi C., Zona C., Orsato A., Shaikh N., Russo L., Nicotra F. (2010). Carbohydrate mimetics and scaffolds: Sweet spots in medicinal chemistry. Future Med. Chem..

[13] Chrysina E.D., Kosmopoulou M.N., Tiraidis C., Kardakaris R., Bischler N., Leonidas D.D., Hadady Z., Somsák L., Docsa T., Gergely P., Oikonomakos N.G. (2005). Kinetic and crystallographic studies on 2-(b-D-glucopyranosyl)-5-methyl-1,3,4-oxadiazole, -benzothiazole, and -benzimidazole, inhibitors of muscle glycogen phosphorylase. Evidence for a new binding site. Protein Sci..

[14] Somsak L., Nagy V., Hadady Z., Docsa T., Gergely P. (2003). Glucose analog inhibitors of glycogen phosphorylases as potential antidiabetic agents: Recent developments. Curr. Med. Chem..

[15] Hadady Z., Tóth M., Somsak L. (2004). *C*-(b-D-glucopyranosyl) heterocycles as potential glycogen phosphorylase inhibitors. ARKIVOC.

[16] Tóth M., Kun S., Bokor É., Benltifa M., Tallec G., Vidal S., Docsa T., Gergely P., Somsák L., Praly J.P. (2009). Synthesis and structure-activity relationships of *C*-glycosylated oxadiazoles as inhibitors of glycogen phosphorylase. Bioorg. Med. Chem..

[17] Sachs G., Shin J.M., Howden C.W. (2006). Review article: the clinical pharmacology of proton pump inhibitors. Aliment. Pharmacol. Ther..

[18] McKellar Q.A., Scott E.W. (1990). The benzimidazole anthelmintic agents-a review. J. Vet. Pharmacol. Ther..

[19] Spasov A., Yozhitsa I., Bugaeva L., Anisimova V. (1999). Benzimidazole derivatives: Spectrum of pharmacological activity and toxicological properties (a review). Pharm. Chem. J..

[20] Beaulieu C., Wang Z., Denis D., Greig G., Lamontagne S., ÓNeill G., Slipetz D., Wang J. (2004). Benzimidazoles as new potent and selective DP antagonists for the treatment of allergic rhinitis. Bioorg. Med. Chem. Lett..

[21] Wan Y., Wallinder C., Plouffe B., Beaudry H., Mahalingam A.K., Wu X., Johansson B., Holm M., Botoros M., Karlén A. (2004). Design, synthesis, and biological evaluation of the first selective nonpeptide AT2 receptor agonist. J. Med. Chem..

[22] Boiani M., González M. (2005). Imidazole and benzimidazole derivatives as chemotherapeutic agents. Mini-Rev. Med. Chem..

[23] Nishida Y., Shingu Y., Mengfei1 Y., Fukuda K., Dohi H., Matsuda S., Matsuda K. (2012). An easy a-glycosylation methodology for the synthesis and stereochemistry of mycoplasma α-glycolipid antigens. Beilstein J. Org. Chem..

[24] Murase H., Moon J.H., Yamauchi R., Kato K., Kunieda T., Yoshikawa T., Terao J. (1998). Antioxidant activity of a novel vitamin E derivative, 2-(a-D-glucopyranosyl)methyl-2,5,7,8-tetramethylchroman-6-ol. Free Radic. Biol. Med..

[25] El-Nezhawy A.O.H., Gaballah S.T., Radwan M.A.A., Baiuomy A.R., Abdel-Salam O.M.E. (2009). Structure-based design of benzimidazole sugar conjugates: Synthesis, SAR and *in vivo* anti-inflammatory and analgesic activities. Med. Chem..

[26] Appel R. (1975). Tertiary Phosphane-Tetrachloromethane, a Versatile Reagent for Chlorination, Dehydration, and P-N Linkage. Angew. Chem. Int. Ed..

[27] Lee J., Nolan T. (1966). Sugar Esters: III. New reagent for deoxyhalo sugar preparation. Can. J. Chem..

[28] Arumugam N., Abdul Rahim A.S., Abd Hamid S., Rosli M.M., Fun H.K. (2010). Ethyl 3-amino-4-[(2-hydroxyethyl)amino]benzoate. Acta Crystallogr. Sect. E: Struct. Rep. Online.

[29] Yet L., Gribble G.W., Joule J.A. (2011). Five-membered ring systems: With more than one N atom. Progress in Heterocyclic Chemistry.

[30] Wright J.B. (1951). The Chemistry of the Benzimidazoles. Chem. Rev..

[31] Gogoi P., Konwar D. (2006). An efficient and one-pot synthesis of imidazolines and benzimidazoles via anaerobic oxidation of carbon-nitrogen bonds in water. Tetrahedron Lett..

[32] Lin S., Yang L. (2005). A simple and efficient procedure for the synthesis of benzimidazoles using air as the oxidant. Tetrahedron Lett..

[33] Navarrete-Vázquez G., Moreno-Diaz H., Aguirre-Crespo F., León-Rivera I., Villalobos-Molina R., Muñoz-Muñiz O., Estrada-Soto S. (2006). Design, microwave-assisted synthesis, and spasmolytic activity of 2-(alkyloxyaryl)-1*H*-benzimidazole derivatives as constrained stilbene bioisosteres. Bioorg. Med. Chem. Lett..

[34] Torres-Gómez H., Hernández-Núñez E., León-Rivera I., Guerrero-Alvarez J., Cedillo-Rivera R., Moo-Puc R., Argotte-Ramos R., Carmen Rodríguez-Gutiérrez M.D., Chan-Bacab M.J., Navarrete-Vázquez G. (2008). Design, synthesis and *in vitro* antiprotozoal activity of benzimidazole-pentamidine hybrids. Bioorg. Med. Chem. Lett..

[35] Kappe C.O., Dallinger D. (2005). The impact of microwave synthesis on drug discovery. Nat. Rev. Drug Discov..

[36] Arumugam N., Abdul Rahim A.S., Wahab H.A., Goh J.H., Fun H.K. (2010). Ethyl 1-(2-hydroxyethyl)-2-*p*-tolyl-1*H*-benzimidazole-5-carboxylate. Acta Crystallogr. Sect. E: Struct. Rep. Online.

[37] Lindhorst T. (2007). Essentials of Carbohydrate Chemistry and Biochemistry.

[38] Demchenko A.V. (2003). 1,2-*cis O*-Glycosylation: Methods, Strategies, Principles. Curr. Org. Chem..

[39] Shingu Y., Nishida Y., Dohi H., Kobayashi K. (2003). An easy access to halide ion-catalytic α-glycosylation using carbon tetrabromide and triphenylphosphine as multifunctional reagents. Org. Biomol. Chem..

[40] Nashed M.A., Anderson L. (1982). Oligosaccharides from “standardized intermediates.” Synthesis of a branched tetrasaccharide glycoside related to the blood group B determinant. J. Am. Chem. Soc..

[41] Cromer R., Spohr U., Khare D.P., LePendu J., Lemieux R.U. (1992). Molecular recognition XII. The binding of the H human blood group determinants and congeners by a lectin of *Galactia tenuiflora*. Can. J. Chem..

[42] Shingu Y., Miyachi A., Miura Y., Kobayashi K., Nishida Y. (2005). One-pot α-glycosylation pathway via the generation *in situ* of alpha-glycopyranosyl imidates in *N,N*-dimethylformamide. Carbohydr. Res..

