# Supplementary material for: Straightforward Synthesis of Novel 1-(2′-α-O-D-Glucopyranosyl ethyl) 2-Arylbenzimidazoles
Source: Molecules. 2012 Aug 17;17(8):9887–99. doi: 10.3390/molecules17089887 (PMC6268058; doi:10.3390/molecules17089887)

Supplementary Materials

^{1}H-NMR spectrum of **6a**

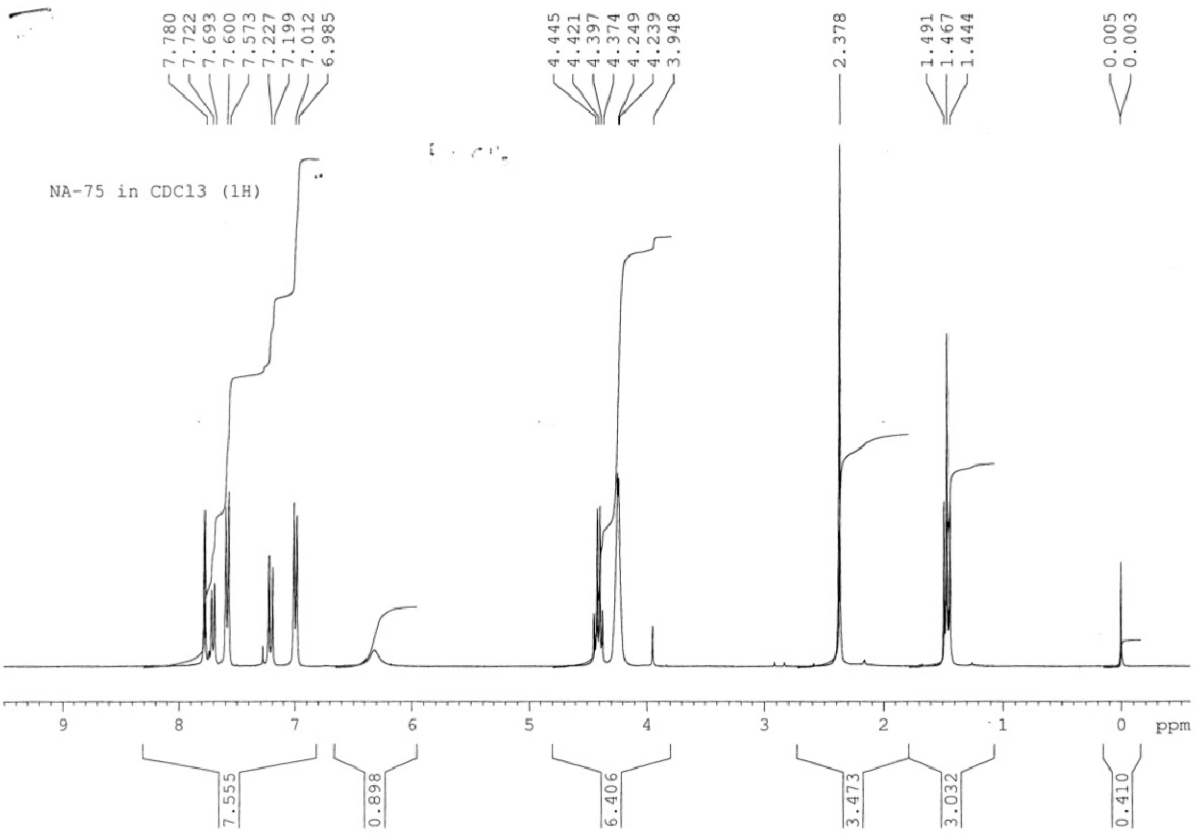

^{13}C-NMR spectrum of **6a**

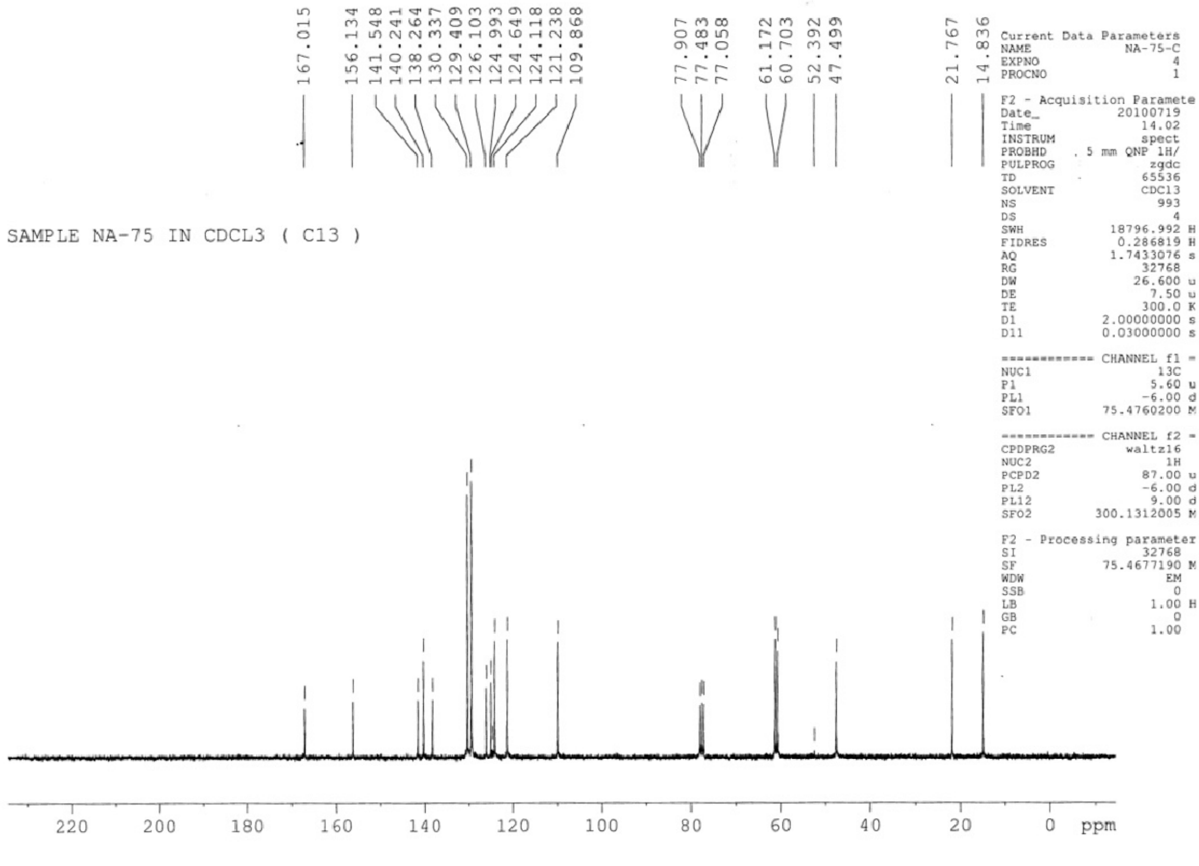

IR spectrum of **6a**

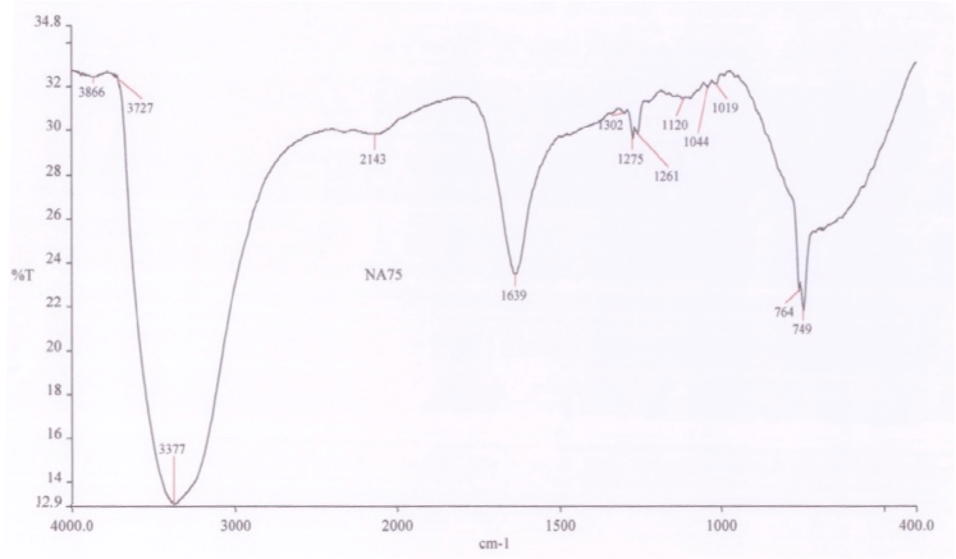

HRMS of **6a**

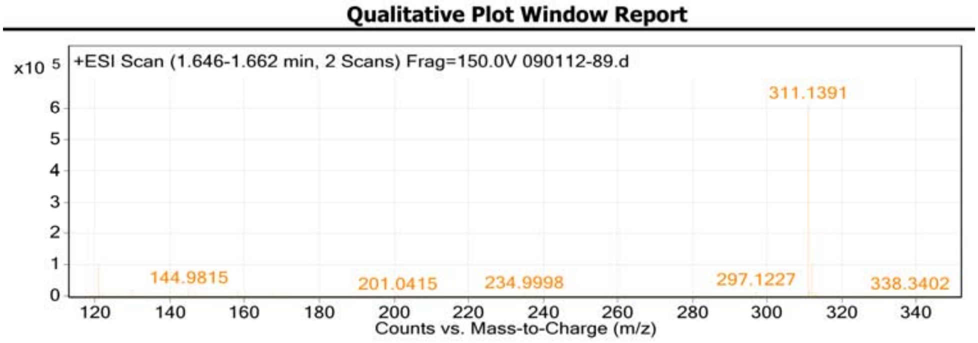

^{1}H NMR spectrum of **6b**

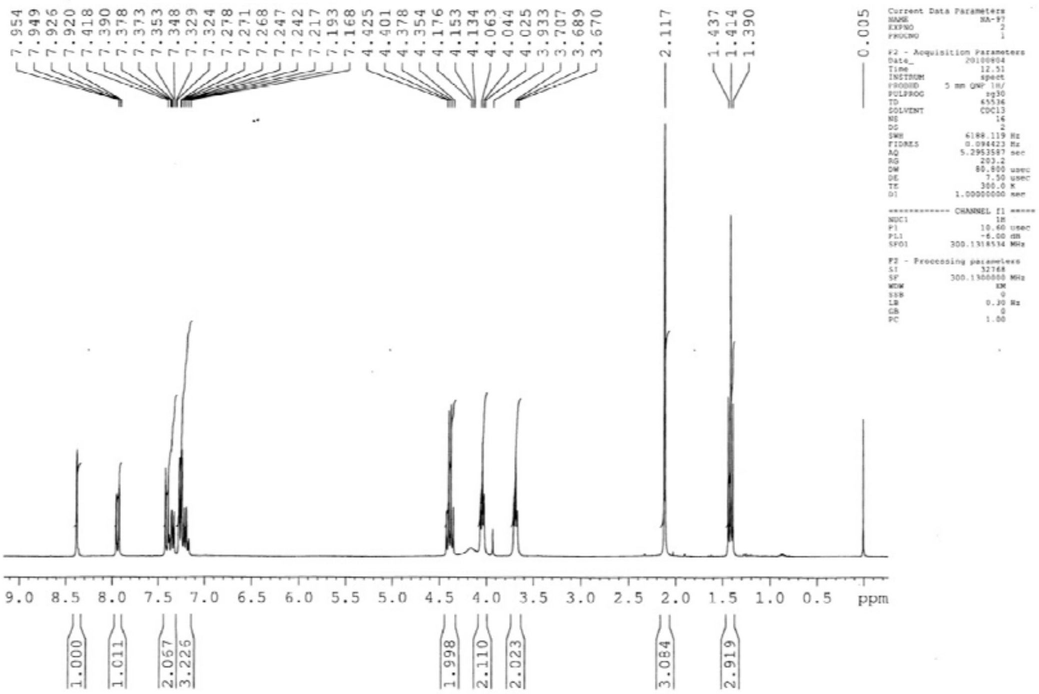

^{13}C-NMR spectrum of **6b**

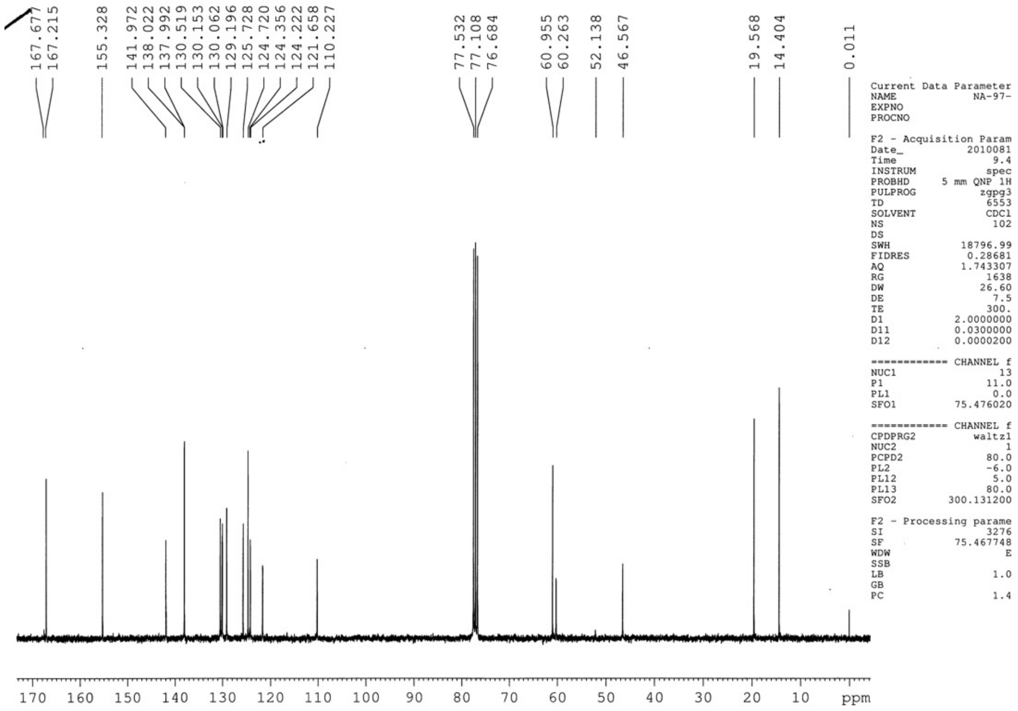

IR spectrum of **6b**

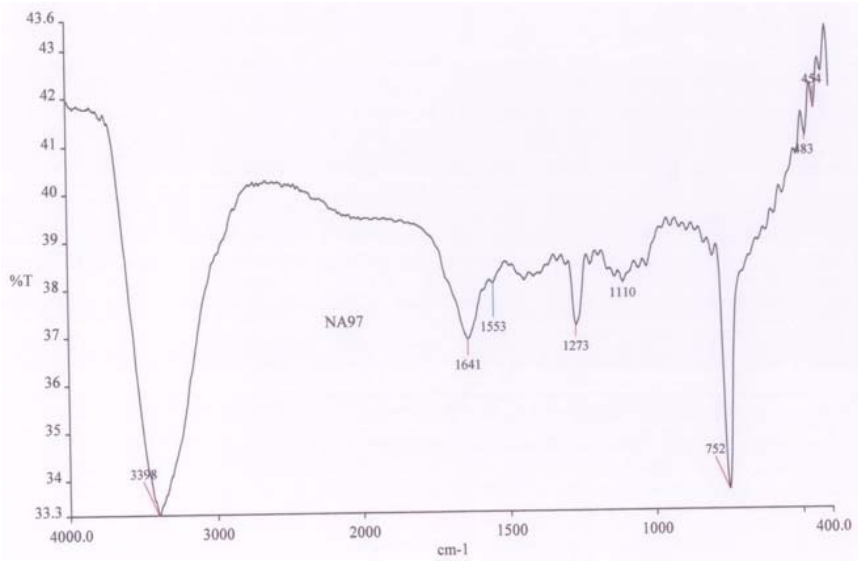

HRMS of **6b**

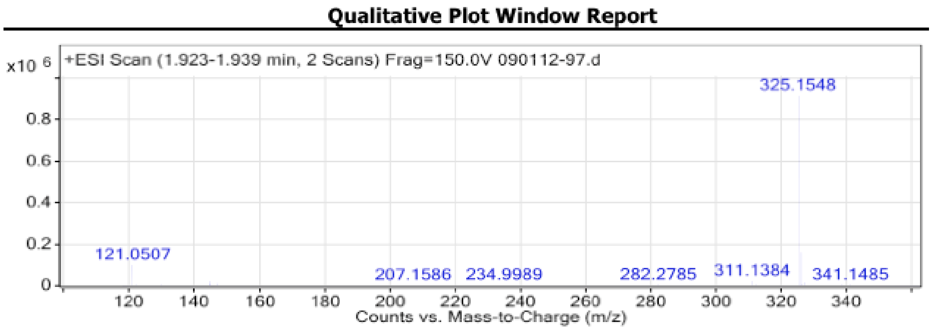

^{1}H-NMR spectrum of **6c**

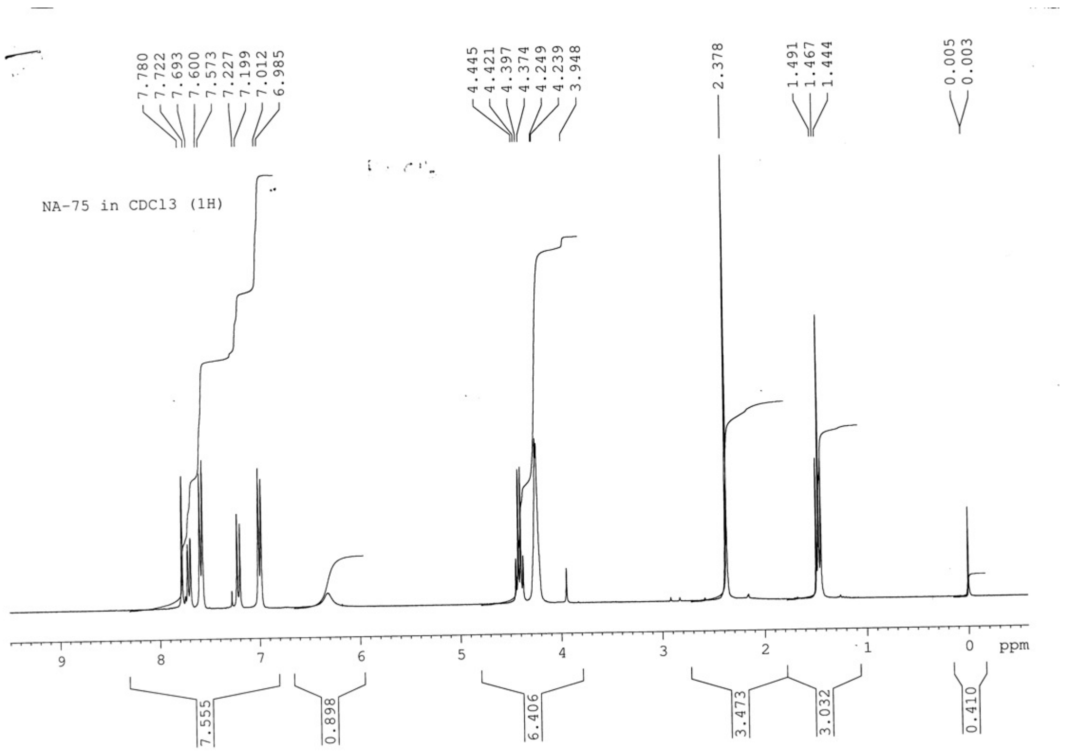

^{13}C-NMR spectrum of **6c**

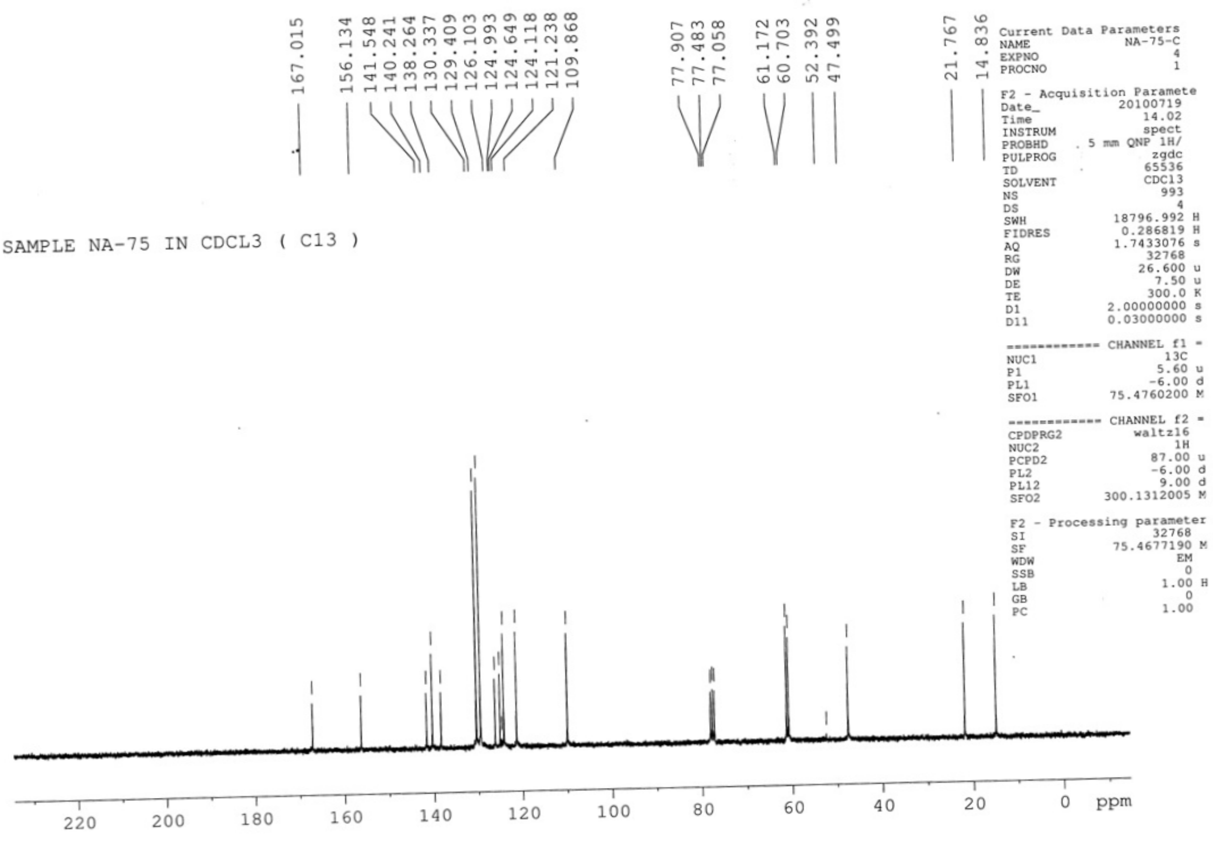

IR spectrum of **6c**

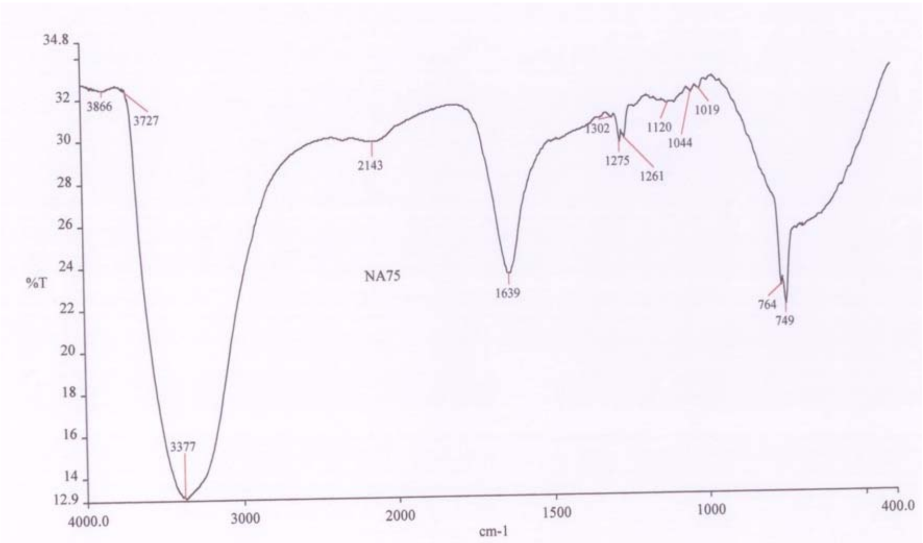

HRMS of **6c**

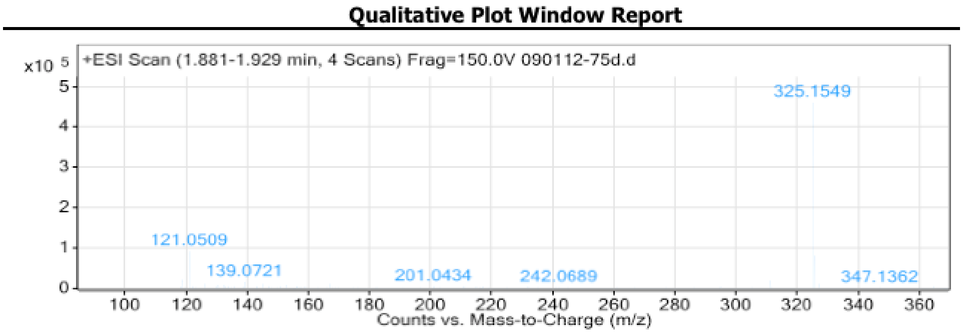

^{1}H-NMR spectrum of **6d**

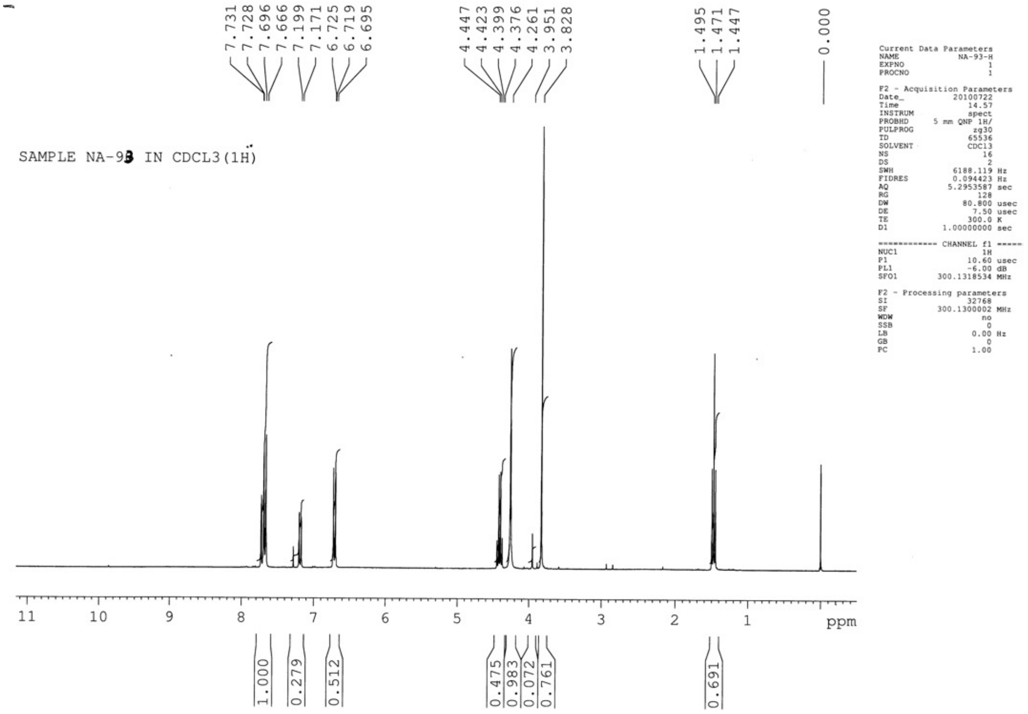

^{13}C-NMR spectrum of **6d**

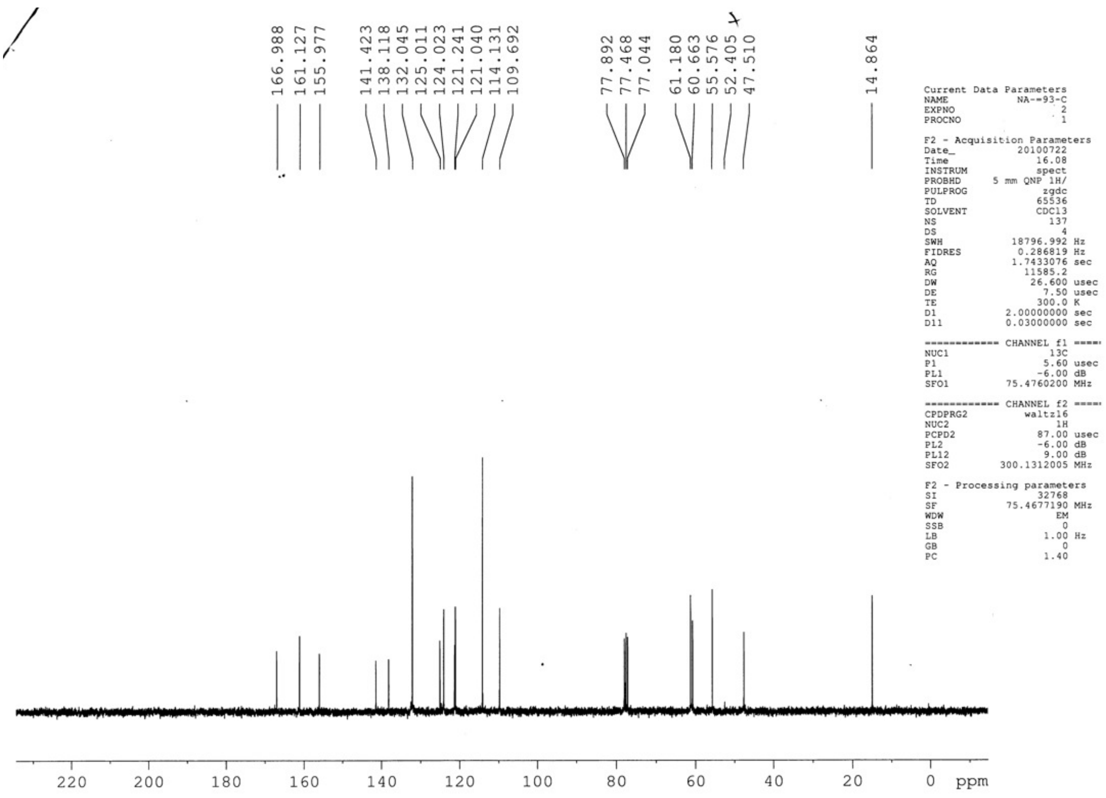

IR spectrum of **6d**

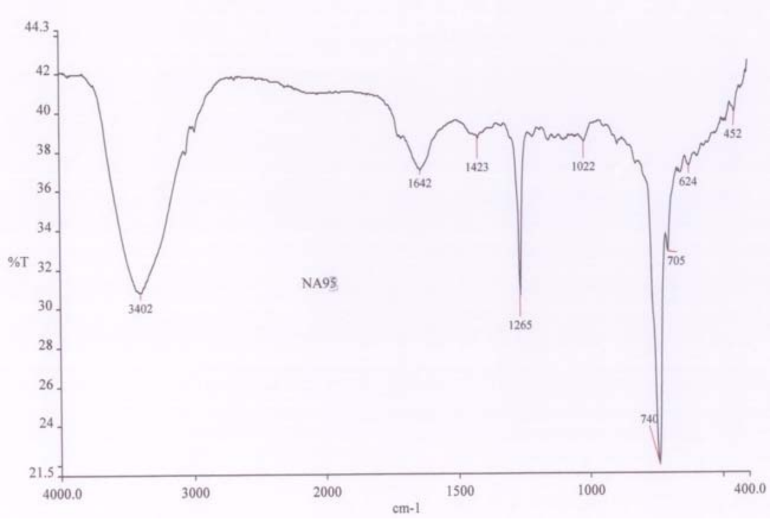

HRMS of **6d**

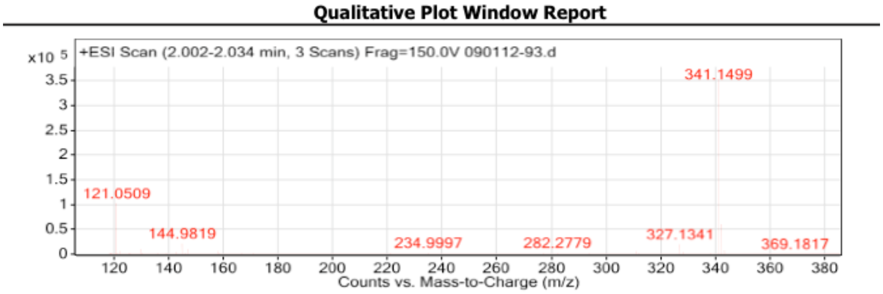

^{1}H-NMR spectrum of **8a**

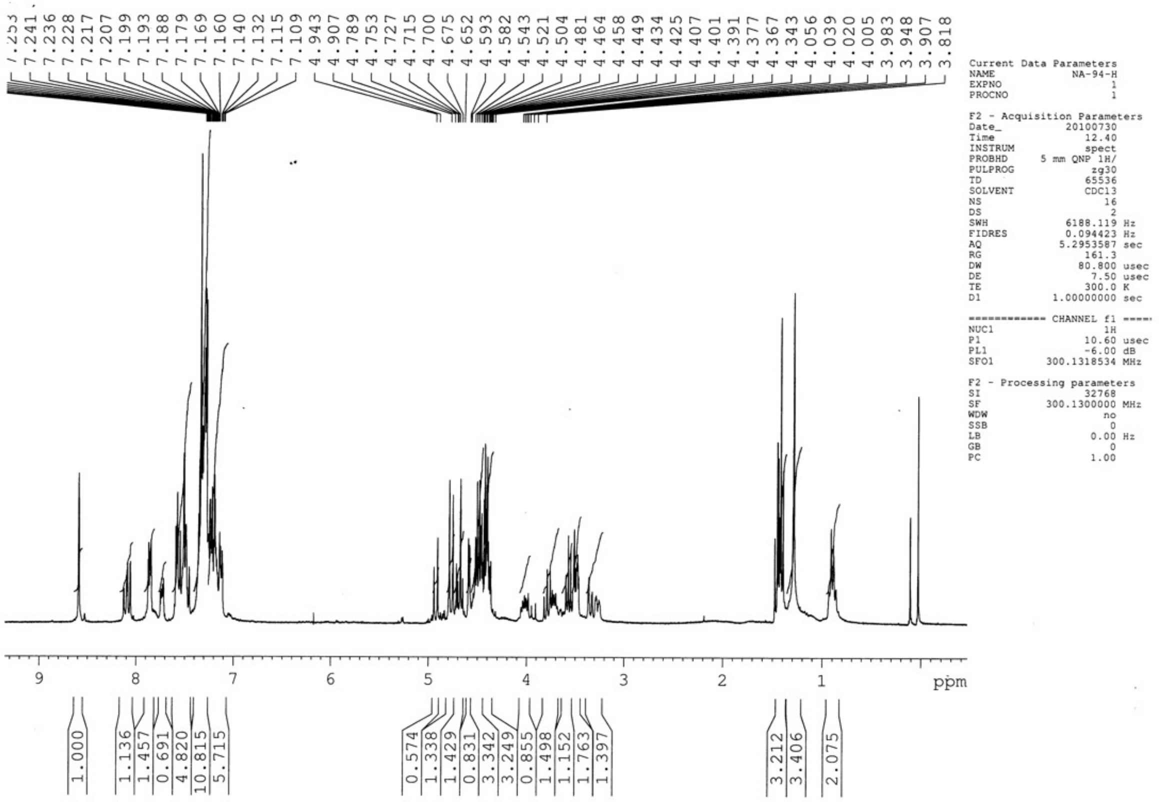

^{13}C-NMR spectrum of **8a**

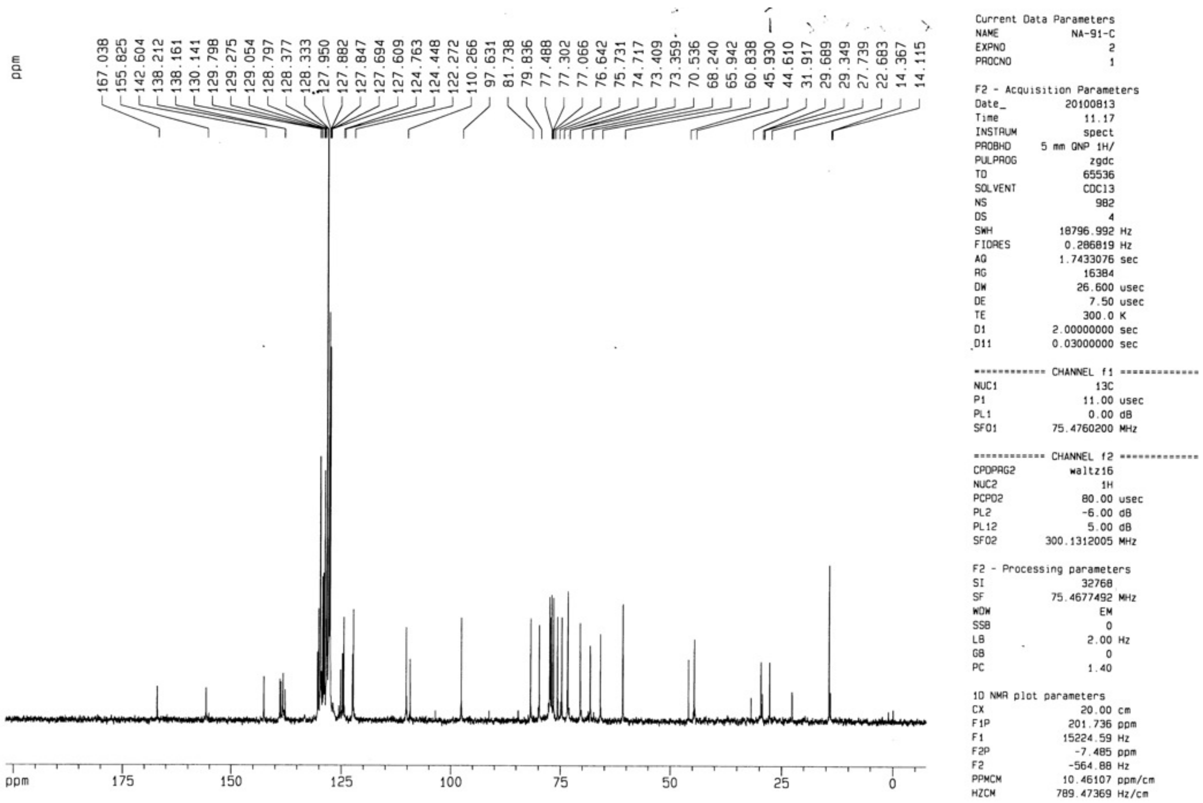

IR spectrum of **8a**

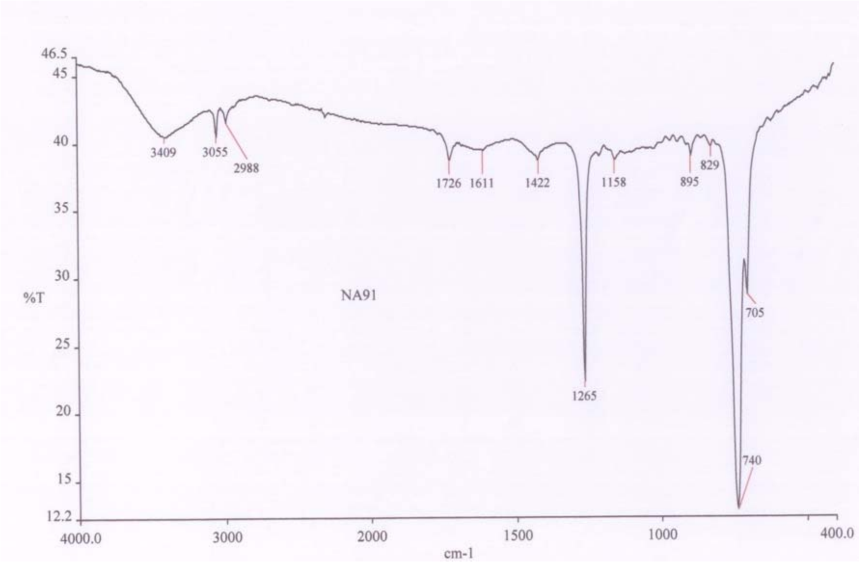

HRMS of **8a**

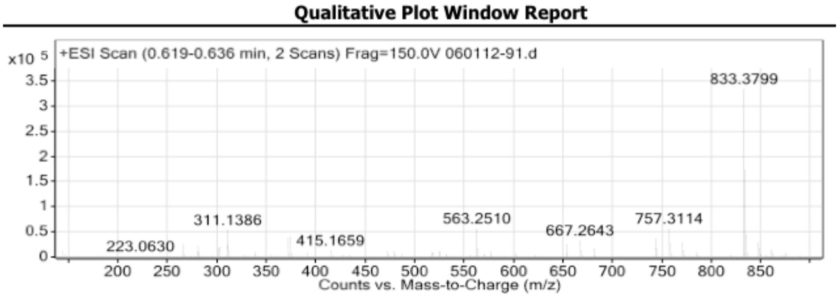

^{1}H-NMR spectrum of **8b**

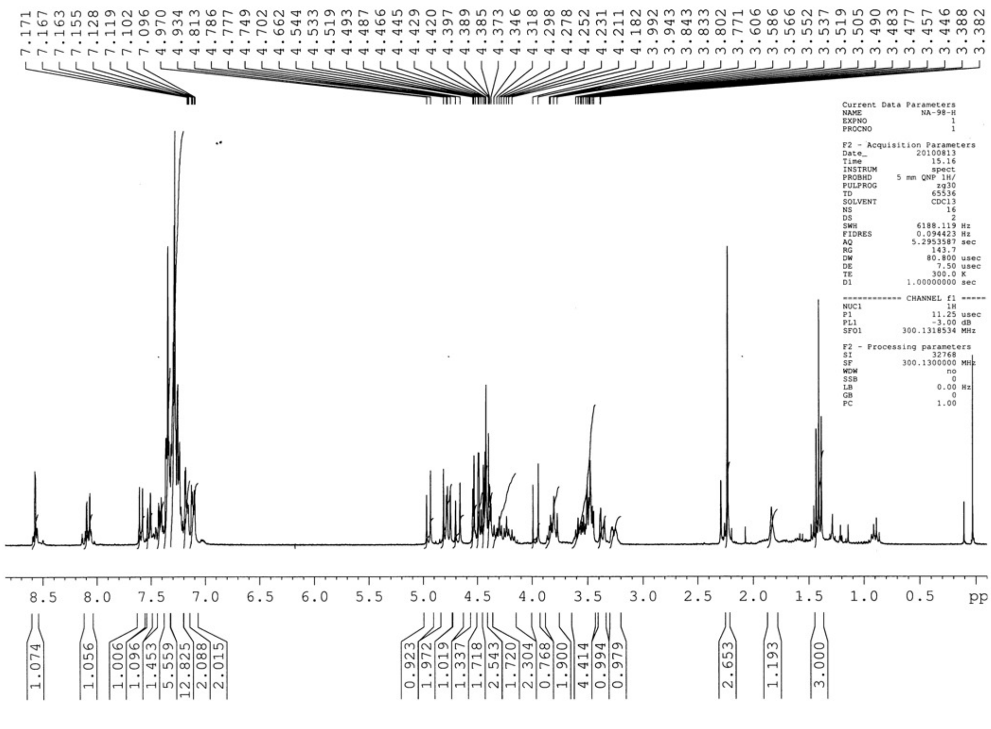

^{13}C-NMR spectrum of **8b**

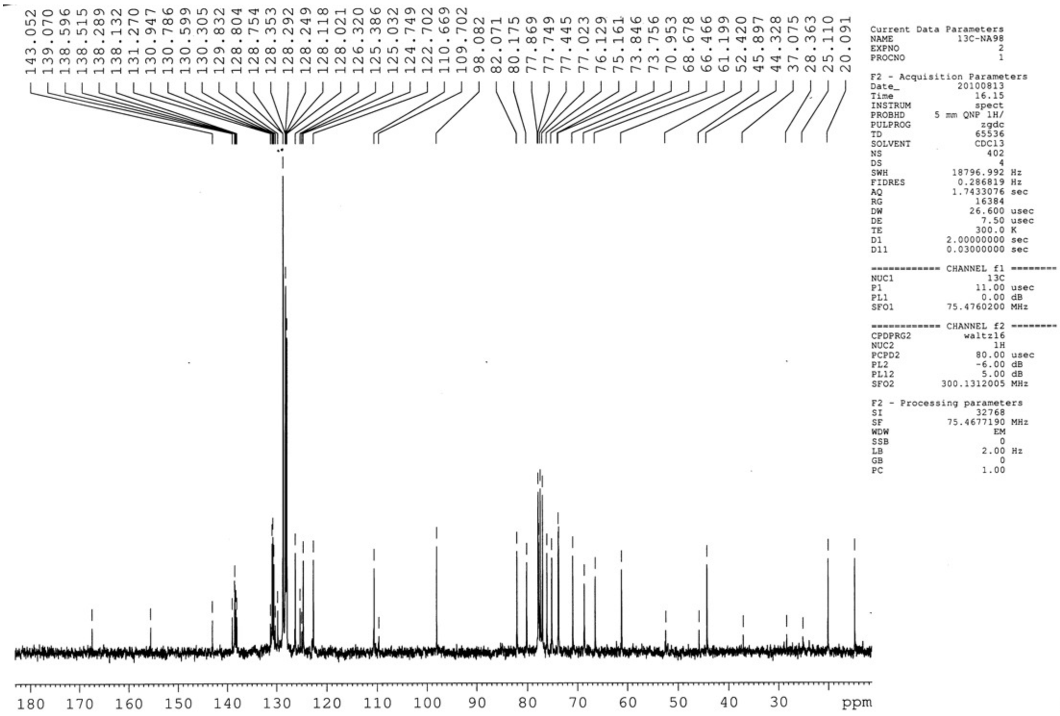

IR spectrum of **8b**

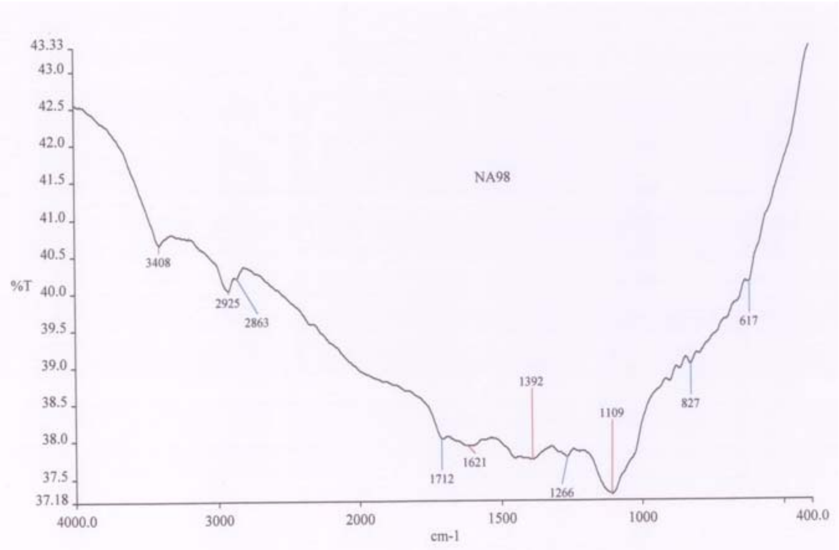

HRMS of **8b**

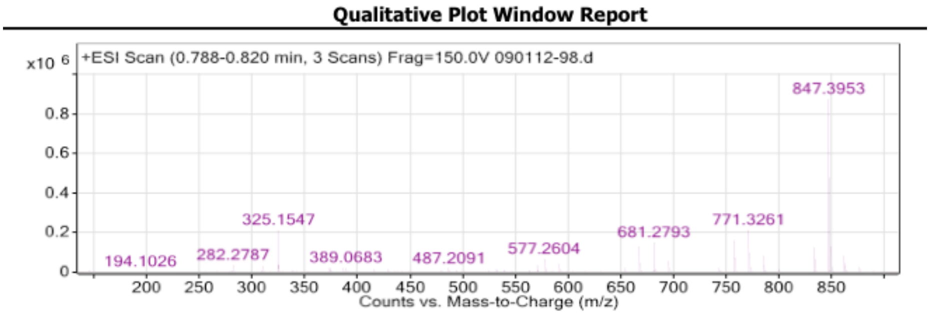

^{1}H-NMR spectrum of **8c**

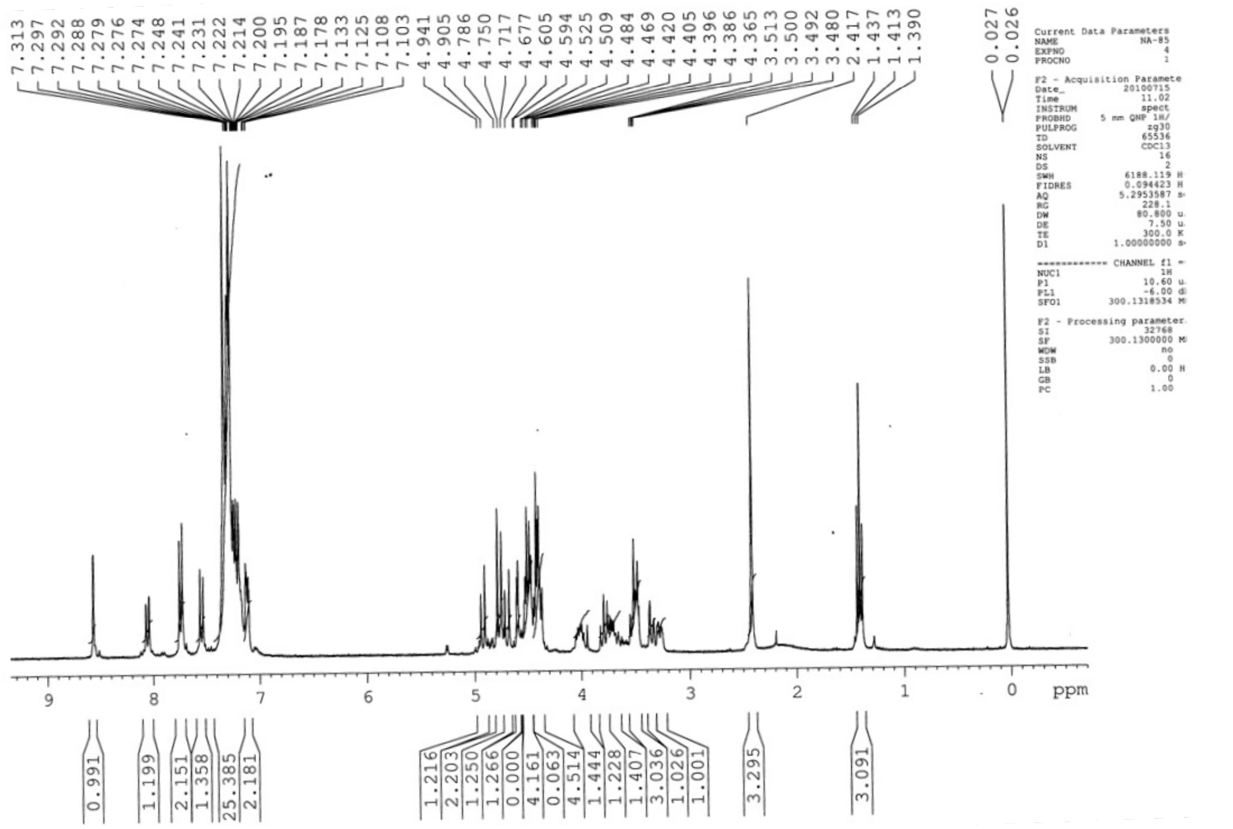

^{13}C-NMR spectrum of **8c**

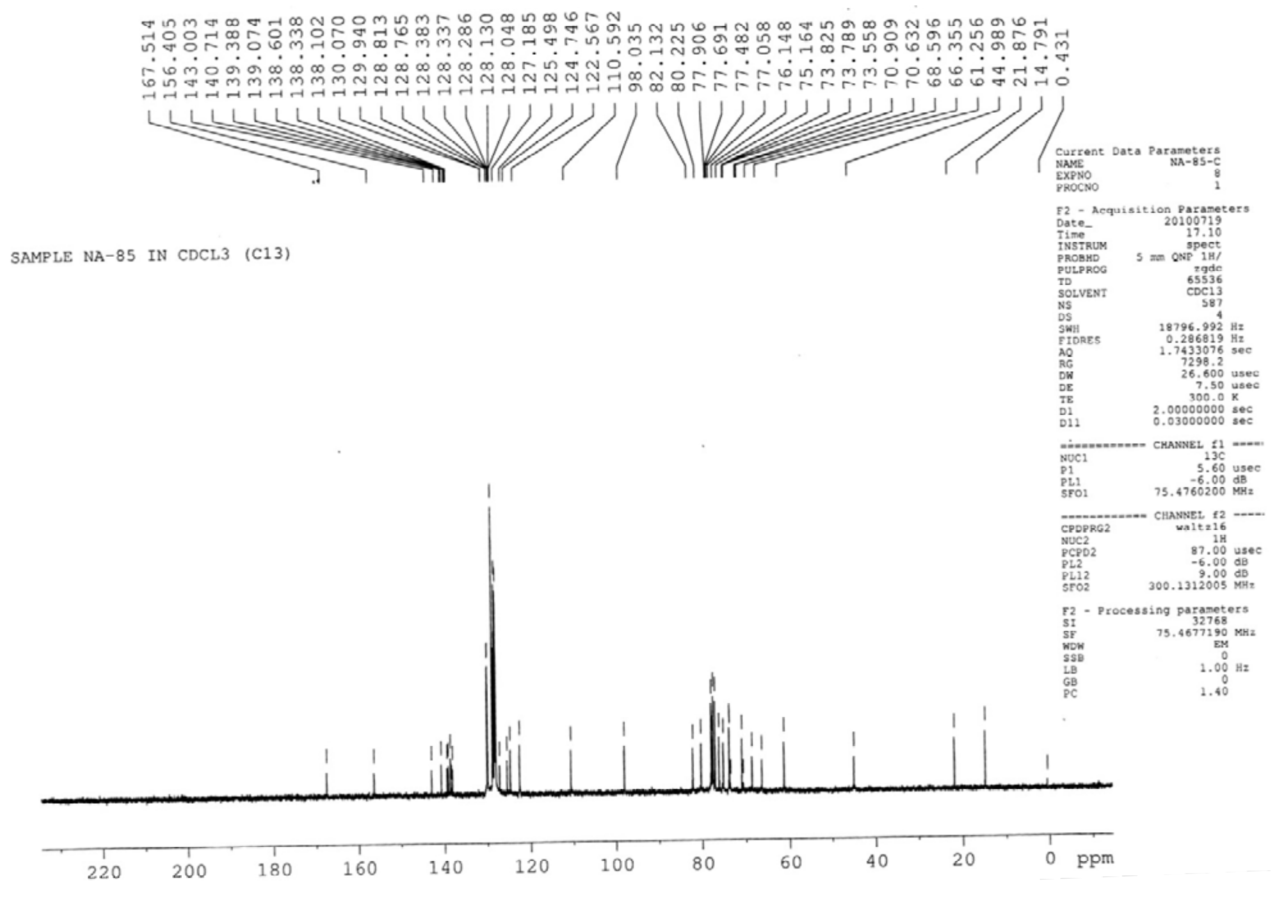

IR spectrum of **8c**

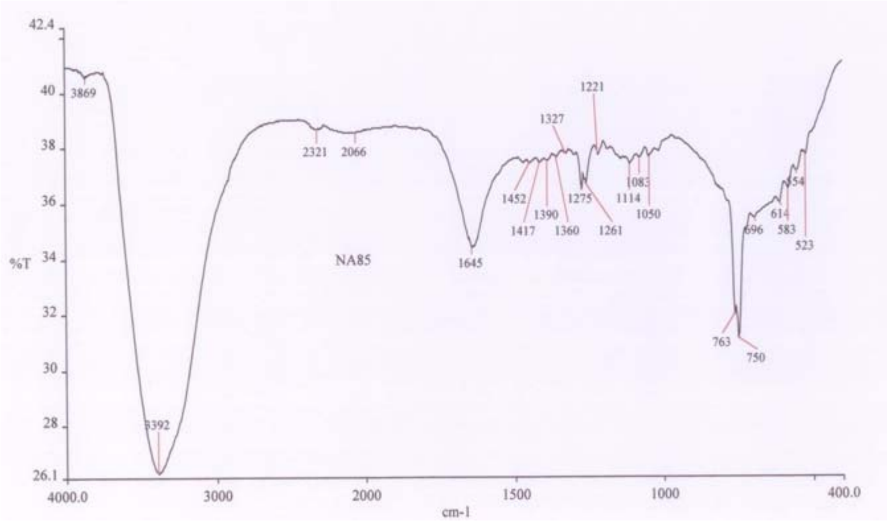

HRMS of **8c**

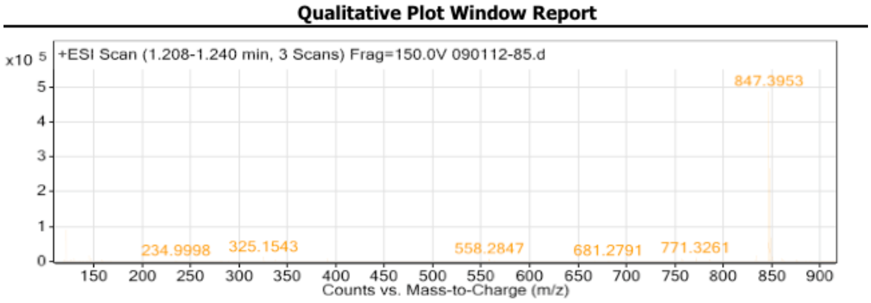

^{1}H-NMR spectrum of **8d**

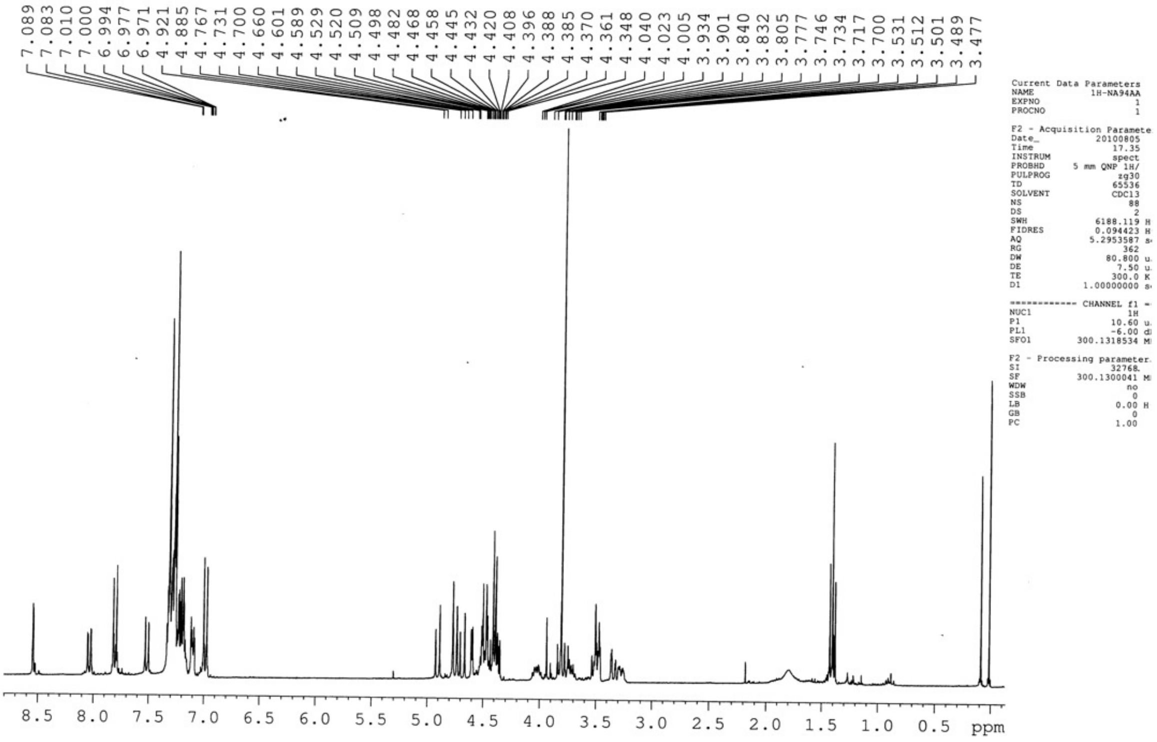

^{13}C-NMR spectrum of **8d**

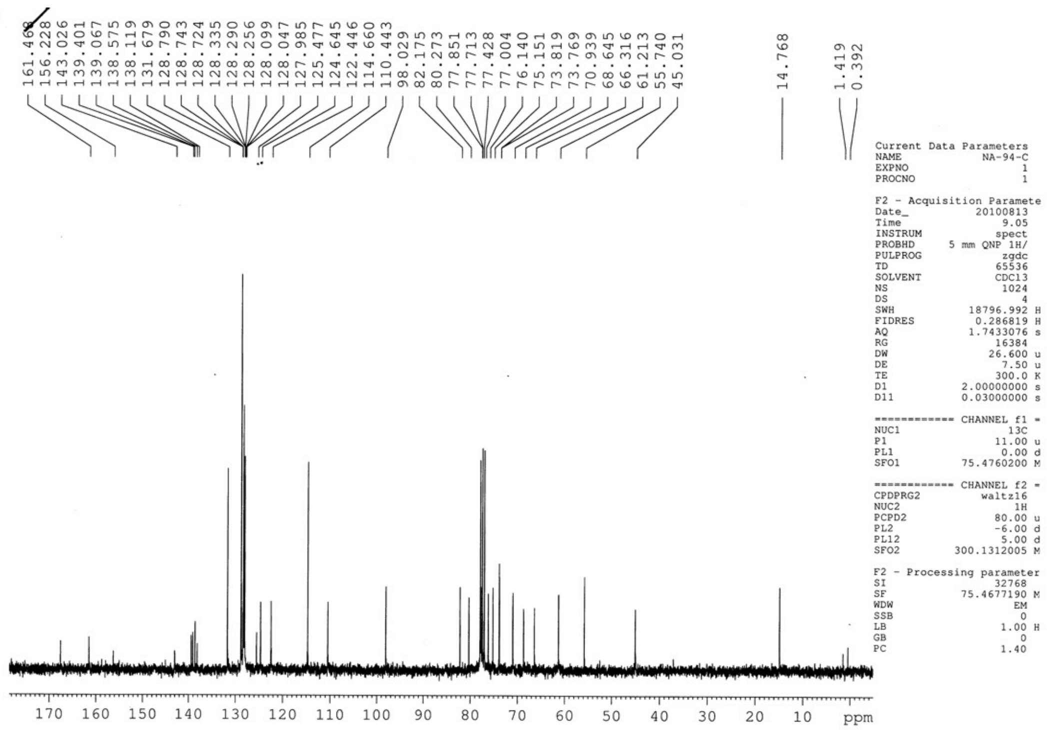

IR spectrum of **8d**

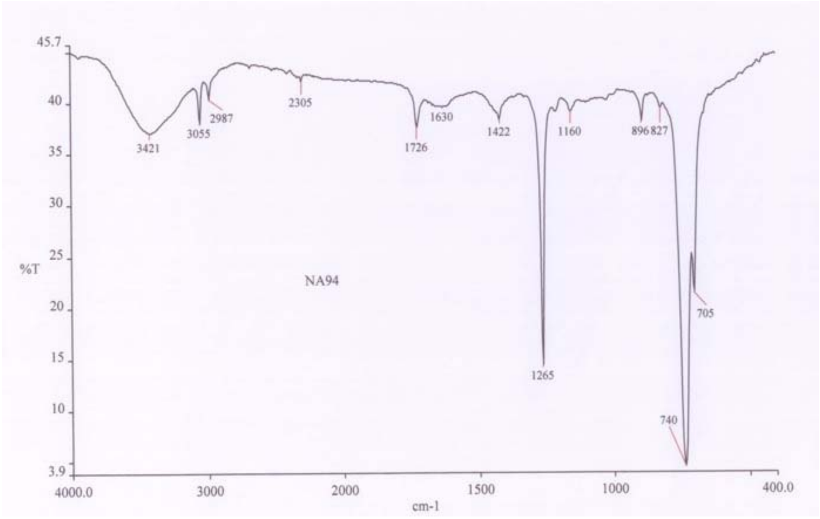

HRMS of **8d**

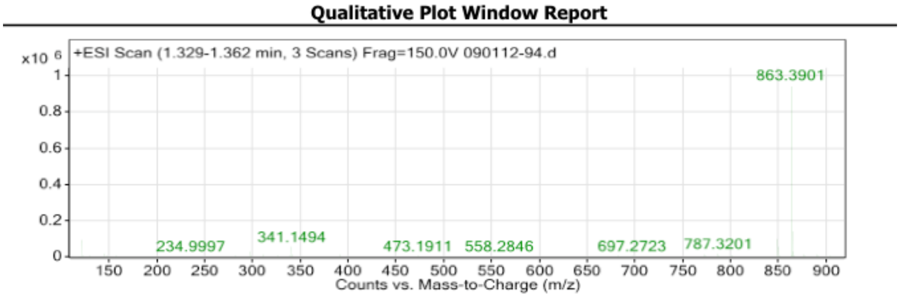

^{1}H-NMR spectrum of **9a**

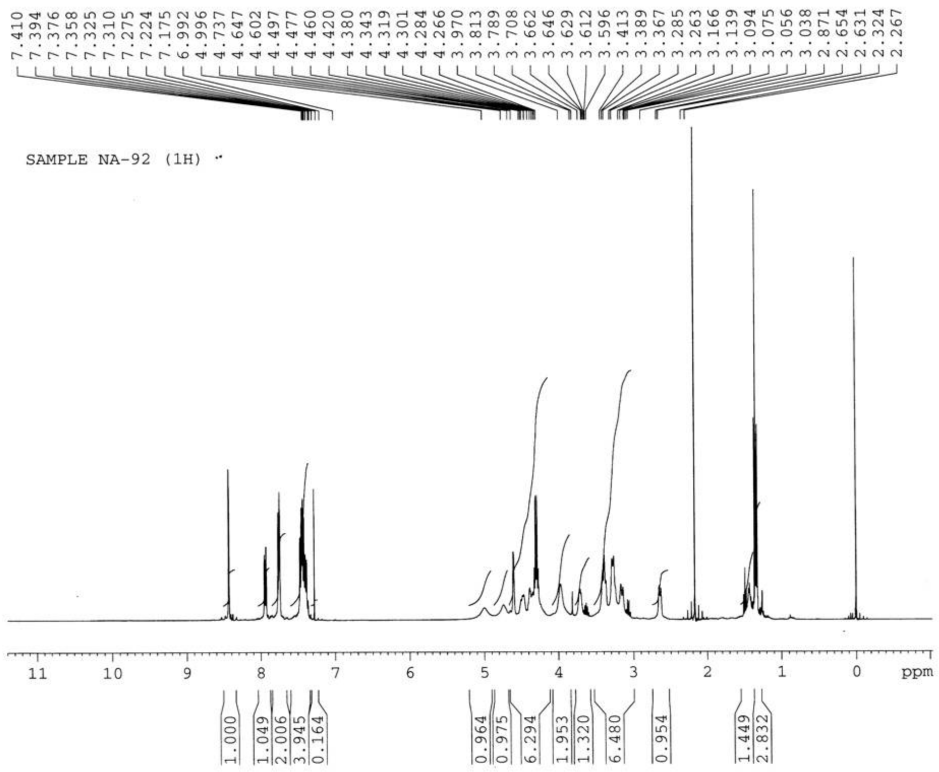

^{13}C-NMR spectrum of **9a**

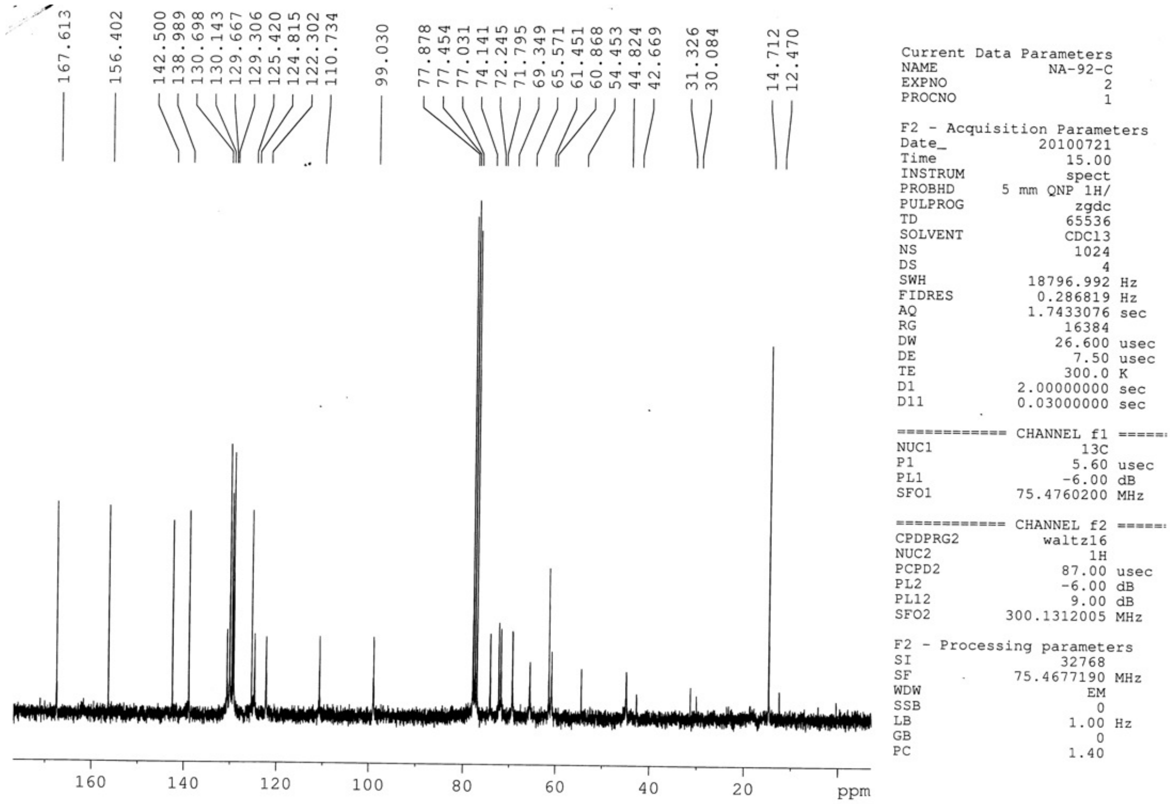

IR spectrum of **9a**

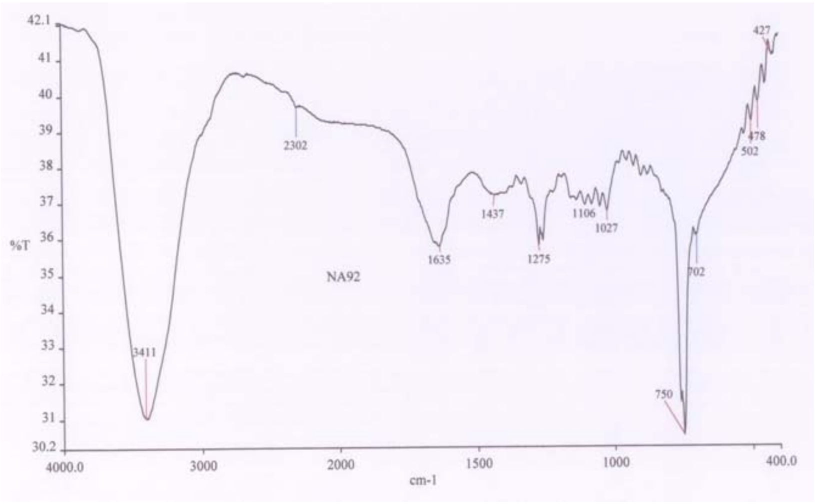

HRMS of **9a**

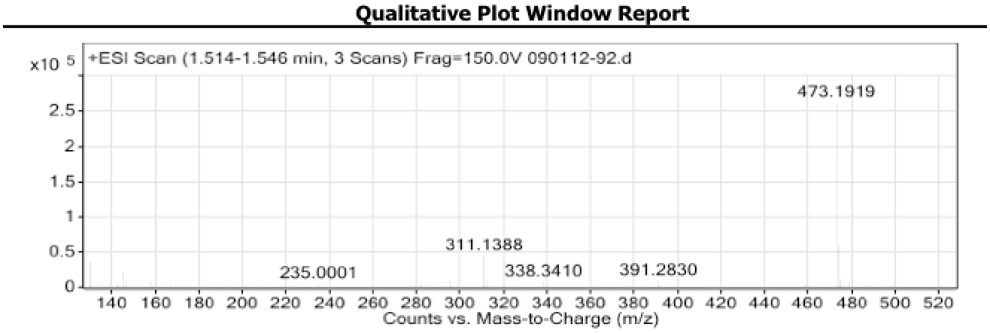

^{1}H-NMR spectrum of **9b**

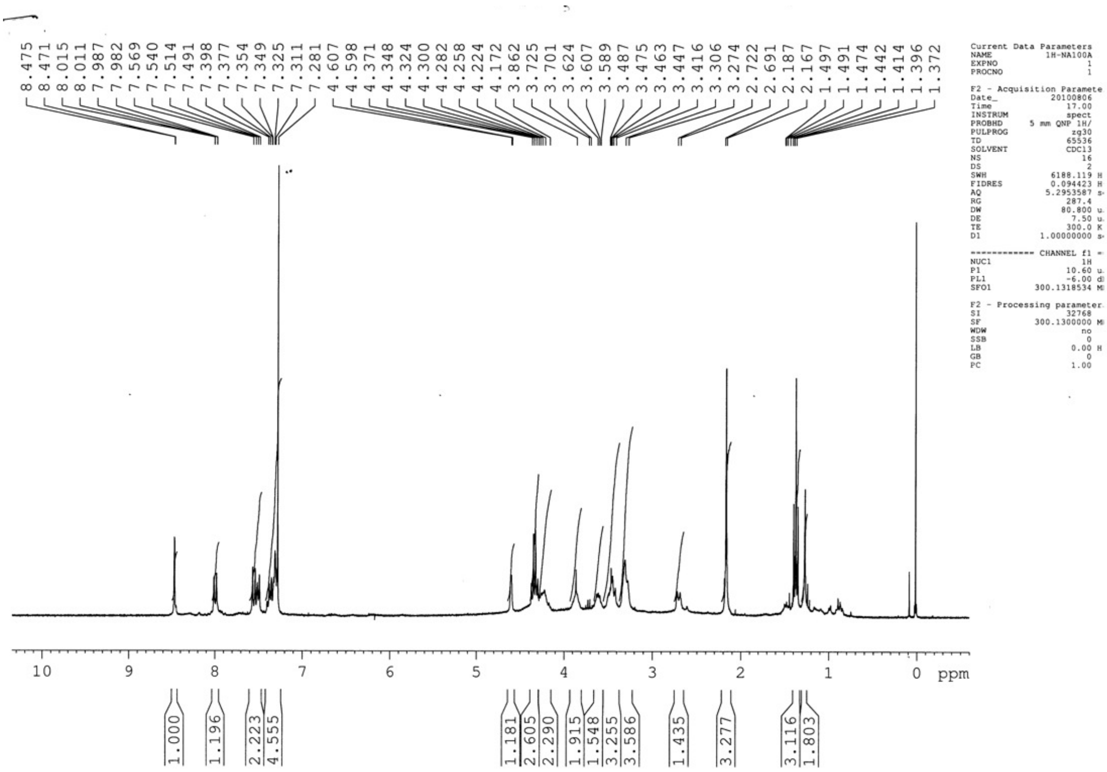

^{13}C-NMR spectrum of **9b**

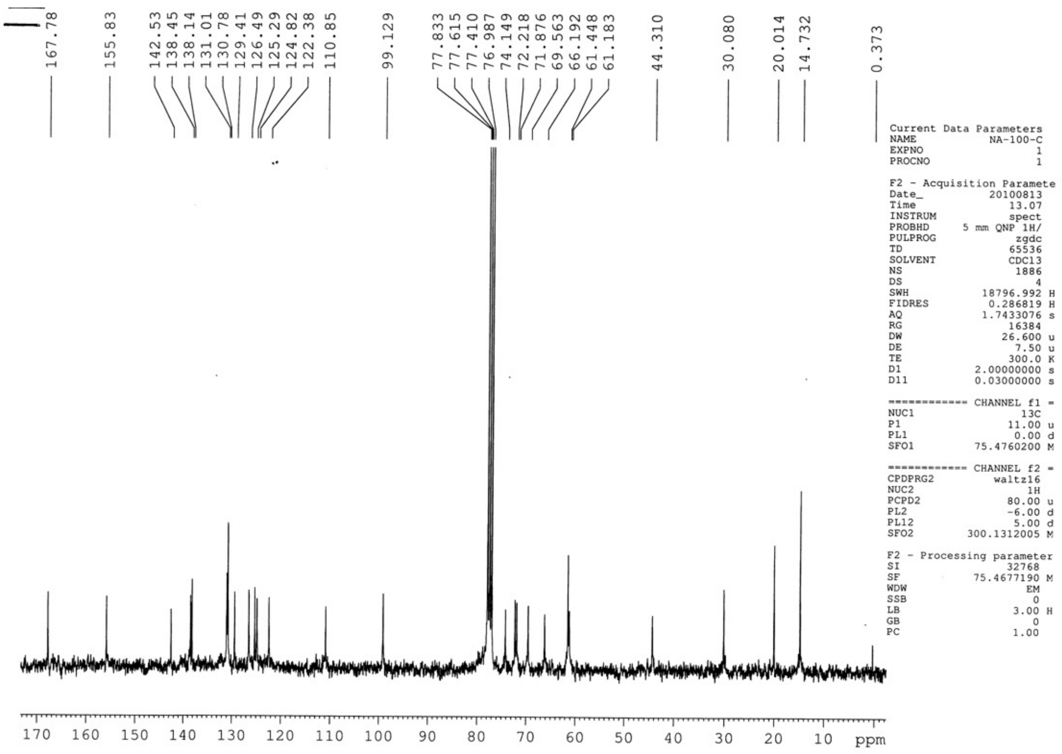

IR spectrum of **9b**

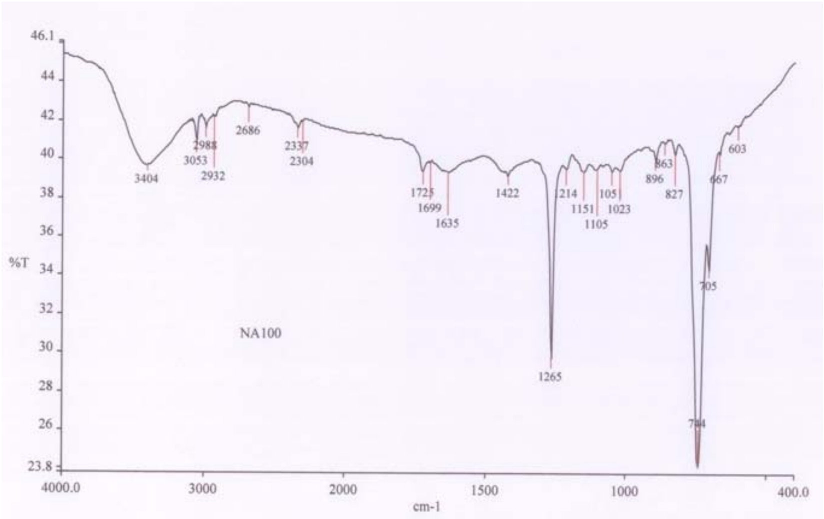

HRMS of **9b**

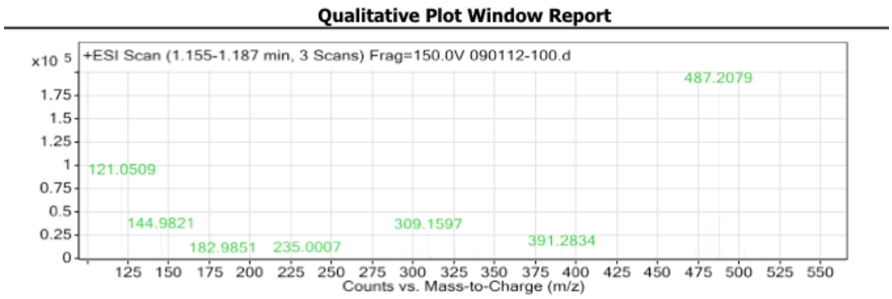

^{1}H NMR spectrum of **9c**

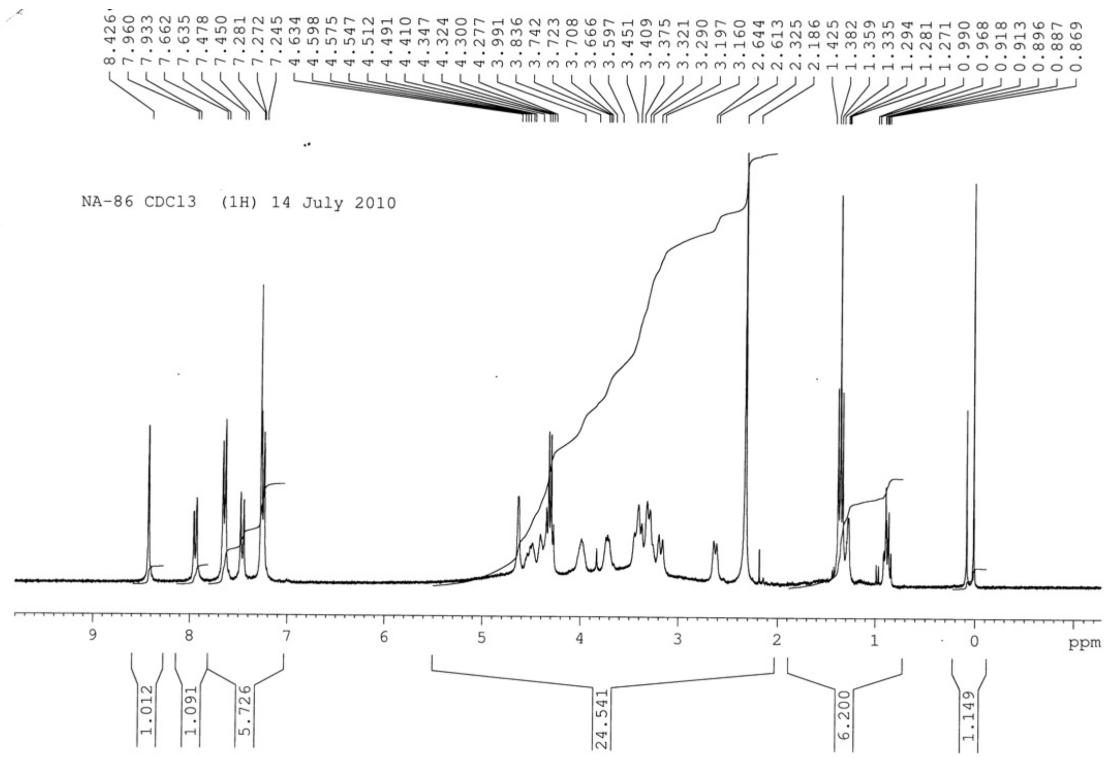

^{13}C-NMR spectrum of **9c**

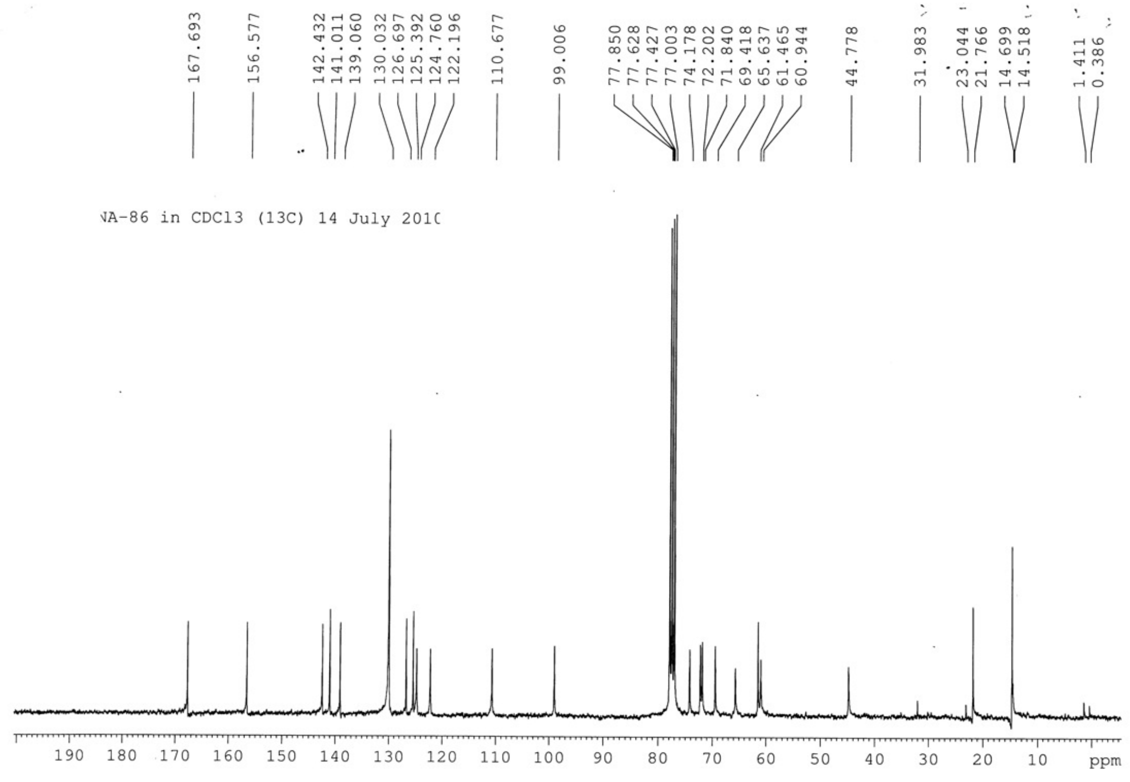

IR spectrum of **9c**

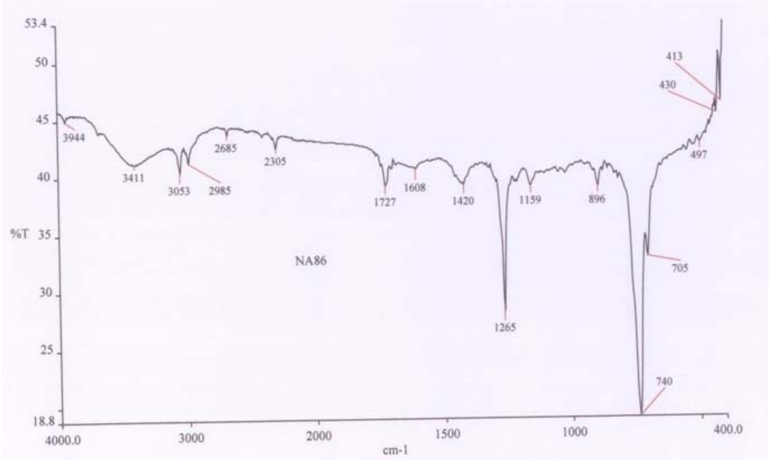

HRMS of **9c**

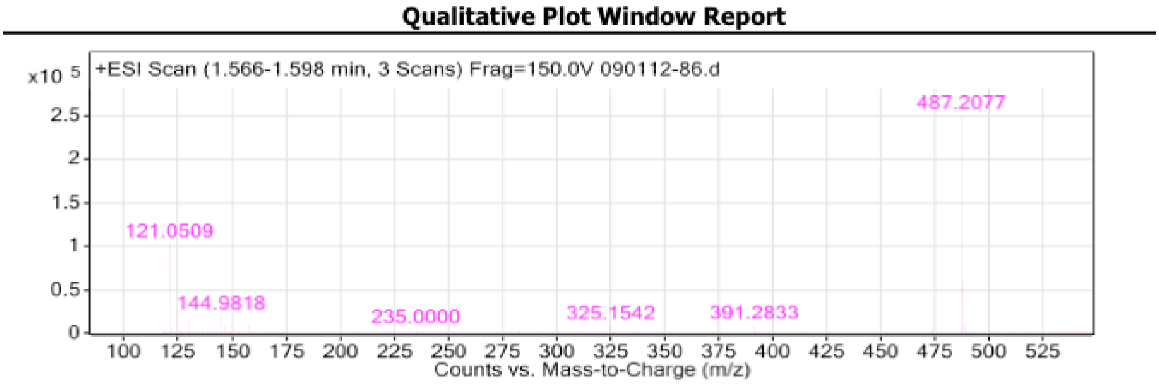

^{1}H-NMR spectrum of **9d**

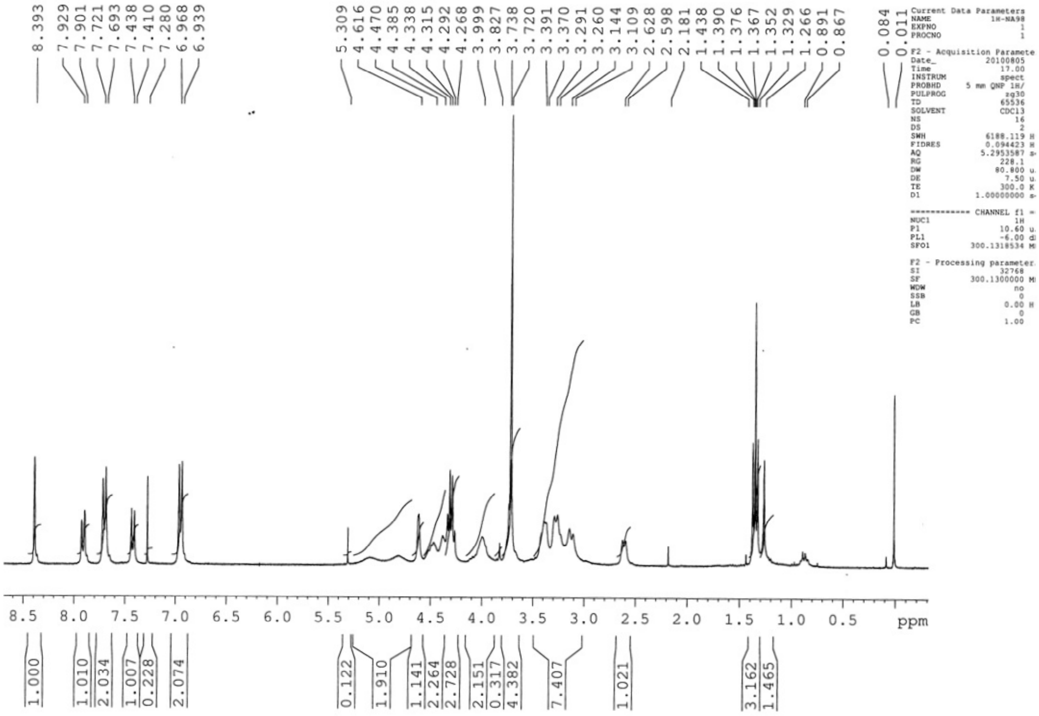

^{13}C-NMR spectrum of **9d**

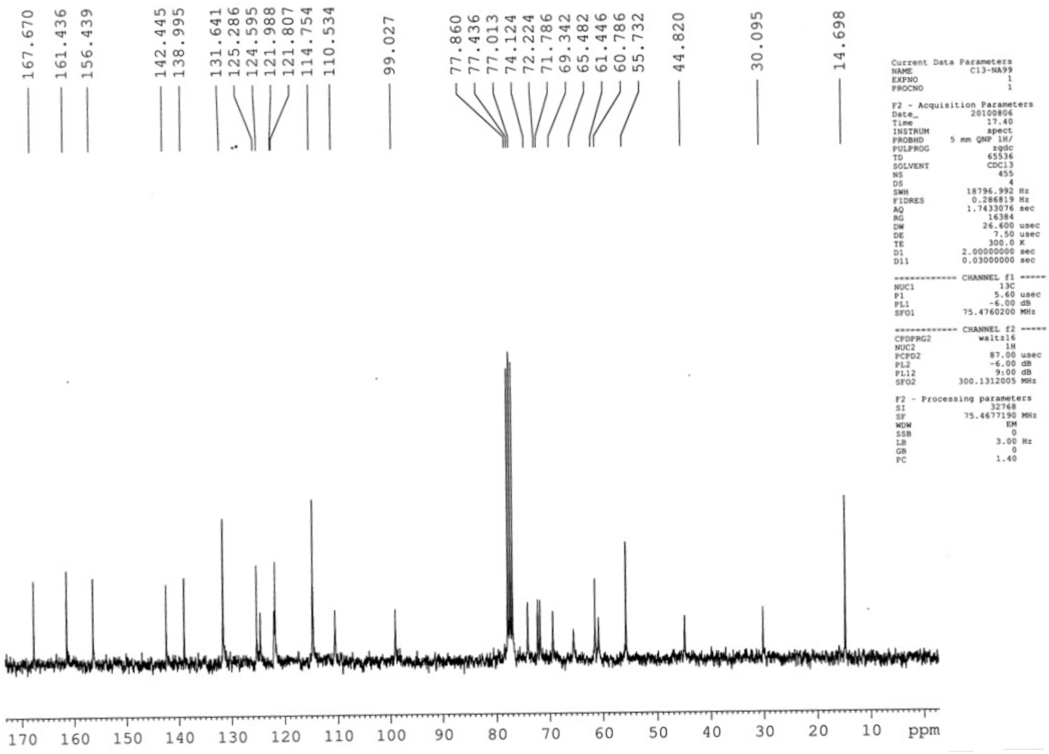

IR spectrum of **9d**

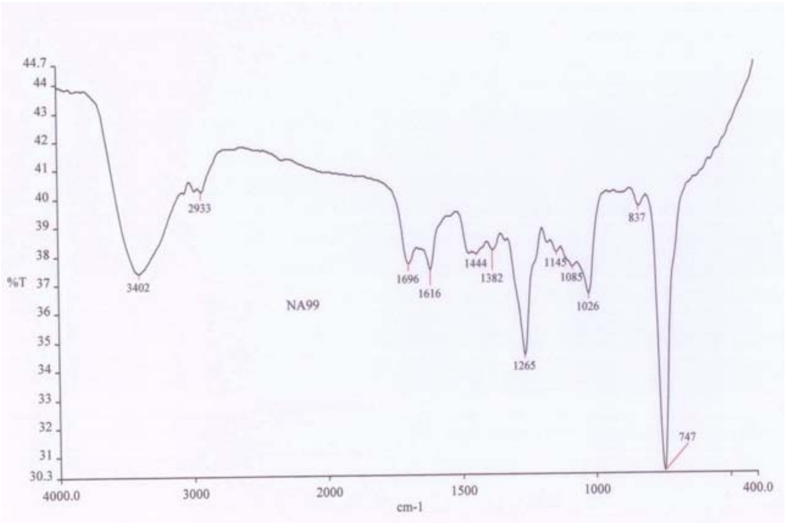

HRMS of **9d**

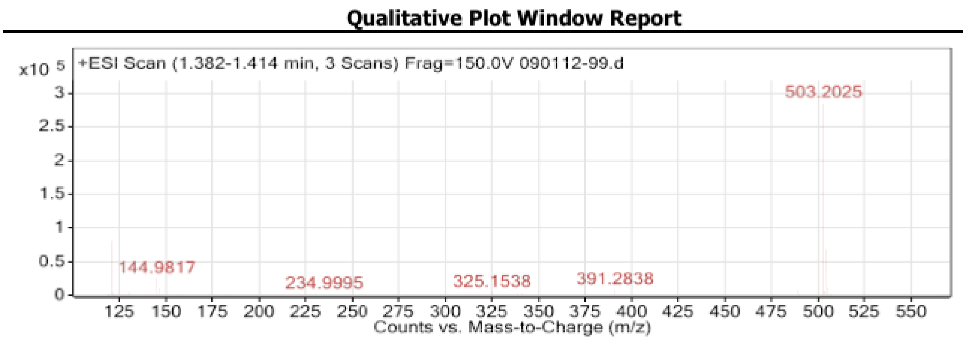

Supplement: Supplementary file 1 [file molecules-17-09887-s001.pdf]
